# Counterintuitive DNA Sequence Dependence in Supercoiling-Induced DNA Melting

**DOI:** 10.1371/journal.pone.0141576

**Published:** 2015-10-29

**Authors:** Rifka Vlijm, Jaco v.d. Torre, Cees Dekker

**Affiliations:** Department of Bionanoscience, Kavli Institute of Nanoscience Delft, Delft University of Technology, Delft, the Netherlands; Florida International University Bimolecular Sciences Institute, UNITED STATES

## Abstract

The metabolism of DNA in cells relies on the balance between hybridized double-stranded DNA (dsDNA) and local de-hybridized regions of ssDNA that provide access to binding proteins. Traditional melting experiments, in which short pieces of dsDNA are heated up until the point of melting into ssDNA, have determined that AT-rich sequences have a lower binding energy than GC-rich sequences. In cells, however, the double-stranded backbone of DNA is destabilized by negative supercoiling, and not by temperature. To investigate what the effect of GC content is on DNA melting induced by negative supercoiling, we studied DNA molecules with a GC content ranging from 38% to 77%, using single-molecule magnetic tweezer measurements in which the length of a single DNA molecule is measured as a function of applied stretching force and supercoiling density. At low force (<0.5pN), supercoiling results into twisting of the dsDNA backbone and loop formation (plectonemes), without inducing any DNA melting. This process was not influenced by the DNA sequence. When negative supercoiling is introduced at increasing force, local melting of DNA is introduced. We measured for the different DNA molecules a characteristic force *F*_char,_ at which negative supercoiling induces local melting of the dsDNA. Surprisingly, GC-rich sequences melt at *lower* forces than AT-rich sequences: *F*_*char*_ = 0.56pN for 77% GC but 0.73pN for 38% GC. An explanation for this counterintuitive effect is provided by the realization that supercoiling densities of a few percent only induce melting of a few percent of the base pairs. As a consequence, denaturation bubbles occur in local AT-rich regions and the sequence-dependent effect arises from an increased DNA bending/torsional energy associated with the plectonemes. This new insight indicates that an increased GC-content adjacent to AT-rich DNA regions will enhance local opening of the double-stranded DNA helix.

## Introduction

Local opening of the DNA helical duplex plays an essential role in DNA-protein interactions in the cell. Not only does the double-stranded DNA (dsDNA) helix have to open up to allow transcription and replication processes, but binding of many proteins requires so-called denaturation bubbles [1–6]. On the other hand, free single-stranded DNA (ssDNA) is rare in cells as it is prone to damage [7]. The balance between dsDNA and denatured ssDNA bubbles is therefore well controlled. Many parameters are known to influence this balance: Binding proteins can locally modify the dsDNA/ssDNA balance by inducing stretching forces and changes to the local supercoiling density (more/less turns within the DNA compared to the relaxed B-DNA) [3,8,9]. Salt concentration, temperature and pH are also known to influence the ssDNA/dsDNA balance, but these are global conditions that are either set by the environment or held constant by the cell [10–12].

This paper addresses the question to what extent the GC content of the DNA sequence contributes to supercoiling or to local melting when DNA is under negative torque. The stability of the double-stranded helix can be estimated from the interaction between the connecting bases of opposite DNA strands. Since the bases A and T mutually interact through 2 hydrogen bonds, and G and C through 3 hydrogen bonds, the G-C base pair forms a stronger bond. Studies in which short linear dsDNA oligomers were heated to melt to ssDNA indeed showed that AT-rich sequences melt at lower temperatures than GC-rich sequences [13,14]. AT-rich DNA sequences are thus expected to exhibit more transient bubble formation than GC-rich sequences, and AT-rich sequences are expected to promote protein binding through transient bubble formation. (De)stabilization of special regions of the genome can thus be programmed within the DNA sequence [15–17].

One important aspect that is not taken into account in temperature-dependent oligomer-melting experiments, is the presence of supercoiling in chromatin. DNA in cells is very long and compacted into large structures where it is not free to rotate towards relaxed B-DNA. *In vivo*, DNA is maintained at 3–9% negative supercoiling [18]. Since B-DNA has a right-handed helicity, the negative supercoiling has a destabilizing effect on the double-stranded helix [7–9,19]. Already low forces of about 0.6pN are known to melt DNA under negative supercoiling densities of a few percent, which are relevant conditions for cellular DNA. DNA melting under negative supercoiling has been extensively studied, but so far comparison between sequences with different GC-content has not been examined experimentally. Instead, the results obtained by sequence-dependent melting induced by temperature have been assumed to be indicative for supercoiling-induced melting as well. Here, we verify this assumption by studying the sequence-dependent stability of DNA molecules under supercoiling densities between -6% and +6%, and stretching forces up to 1.2pN.

Using magnetic tweezers we measure the extension of single DNA molecules under varying supercoiling densities. Positive supercoiling is absorbed by the formation of plectonemes; hence, the introduction of extra turns to the DNA results in a length decrease. Under negative supercoiling and forces below 0.5pN, introduction of more negative turns similarly results in a length decrease due to plectoneme formation. Above 0.5pN, however, there is a competition between plectoneme formation and DNA melting. By comparing the DNA extension at negative supercoiling densities with the extension at comparable positive supercoiling densities, the length increase due to melted DNA can be accurately measured. In the force-regime with a co-existence of plectonemic and locally melted DNA, the transition between these two topological states also increases the noise level of the length measurements. The characteristic melting force *F*_*char*_ can therefore independently be determined from two types of measurements, viz. the length of the molecule and its standard deviation (*std*). We carried out an extensive set of experiments that measured these quantities for DNA with six different DNA sequences with a varying GC content between 38% and 77%. From temperature-dependent melting experiments, an increase in GC-content was expected to result in an increased melting force *F*_*char*_. Surprisingly however, the opposite result was found! *F*_*char*_ was found to be lower (~0.56pN) for DNA with 77% GC than for DNA with 38% GC (~0.73pN). This result can be understood from the realization that the application of negative supercoiling densities of a few percent will only melt a few percent of the base pairs. As the melting energy of AT base pairs is smaller than that of GC base pairs, the denaturation bubbles will, independent of average GC content, localize in the AT-dense parts of the sequences. As the characteristic melting force, which characterizes the transition from plectonemes to melted DNA, depends on a balance between the melting energy and the bending energy associated with the plectonemes, an increasing bending/torsional energy for an increasing GC content of the DNA can lower the characteristic melting force for GC-rich sequences.

## Materials and Methods

**Fig 1:**
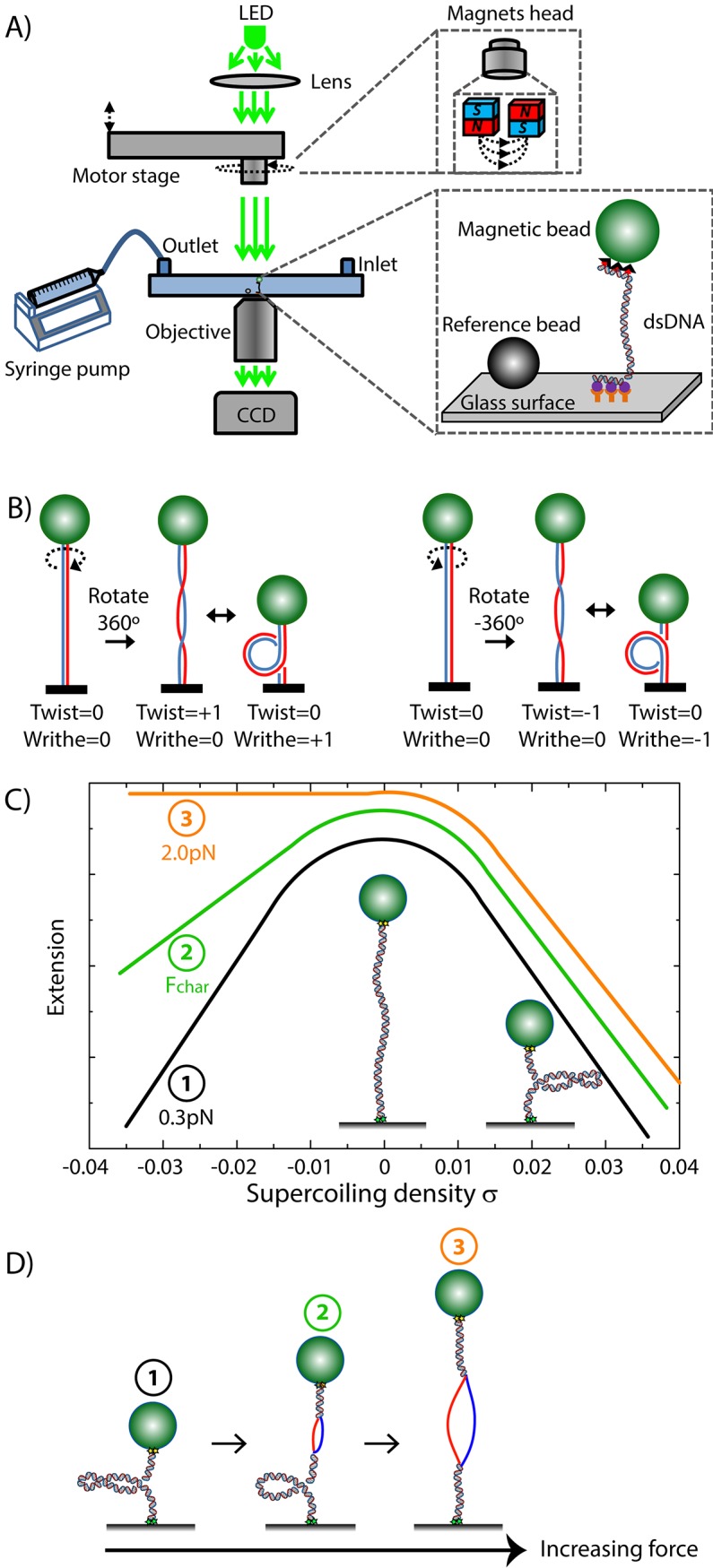
Single-molecule magnetic tweezers. (A) Setup for magnetic tweezers experiments. The lower inset shows how a double-stranded DNA (dsDNA) molecule with multiple DIG-labels at one end is tethered to an anti-DIG-coated glass surface of a flow-cell. The other end of the DNA has multiple biotinlabels and is attached to a streptavidin-coated superparamagnetic bead. Next to the tether is a non-magnetic bead attached to the surface that functions as reference bead for drift correction. Above the flow cell is a holder containing two block magnets which induce a magnetic field. By changing the height of the magnets head with the motor stage, the applied stretching force on the DNA can be varied. When rotating the magnets head, the magnetic bead also rotates, changing the supercoiling density of the dsDNA. Buffer exchange can be done by pipetting new buffer into the fluid inlet, and removing the old buffer by turning on the syringe pump. Green LED light is made parallel by a lens and illuminates the beads. The bead images are recorded by a CCD camera. (B) Illustration of supercoiling. In a rotationally constrained dsDNA molecule, the linking number *L*_*k*_ is constant when no external turns are applied. Linking number is a sum of twist (*Tw*) and writhe (*Wr*), which are interchangeable. The left panel illustrates how two parallel strands are rotated by one positive turn. The total linking number thus goes from 0 to 1. Since twist and writhe are interchangeable, both the image with the intertwined backbones, as well as the image with the parallel backbones and one positive plectoneme, are possible topological DNA states after a linking number change of +1. The right panel shows a similar result after one negative turn. In a rotationally constrained molecule, the induced rotations *ΔLk* thus can be absorbed in a change in helicity of the dsDNA backbones (*Tw*) and/or in a change in the number of plectonemes (*Wr*), *ΔLk* = *ΔTw* + *ΔWr*. (C) Rotation curve. At constant force below 0.6pN, the DNA extension is maximal when no external rotations are applied (supercoiling density *σ* = 0). When positive/negative turns are applied, initially the induced linking number is first absorbed by over/underwinding of the DNA backbone (twist). After a certain buckling point, extra positive/negative turns are no longer absorbed by twist, but by the formation of plectonemes (writhe) which reduce the extension (black curve). For intermediate forces (0.6-5pN), induced positive supercoiling still forms plectonemes (right side of all three curves). Since the dsDNA backbone has a positive helicity, negative applied turns have a destabilizing effect. Negative applied turns in the intermediate force regime of 0.6-5pN therefore have a different effect on the extension. Instead of plectoneme formation, part of the applied turns are absorbed by opening up of the dsDNA backbone into two single DNA strands, i.e. ‘melting’ of the DNA (curves 2 and 3). (D) Illustration of the force-induced transition from plectonemic to melted DNA. Cartoons (1), (2), and (3) are at 0.3pN, the critical melting force (defined as *F*_*char*_ ~0.6pN), and 2pN, respectively, as in Panel C).

In the magnetic tweezers, one monitors the end-to-end length (*Z*) of a torsionally constrained dsDNA molecule that is tethered between a surface and a bead in a flow cell (Fig 1A). For drift correction, reference beads are flushed in (Bang Laboratories, Carmel, IN). We illuminated the flow cell by LED (Cree® XLamp® XP-E Green) and imaged the beads by a CCD camera (Dalsa FA-20-01M1H) at 100Hz using a home-built inverted microscope (60x final magnification; used objective is Nikon 50X Oil Plan Achromat objective, NA 0.9). By placing a pair of external magnets (separated by 1mm from each other; Supermagnete, W-05-N, 5mm, Neodymium) above the bead, we applied a stretching force (Fig 1A). By adjusting the height of the magnets, the applied stretching force was regulated. Upon rotating the magnets within the x-y plane, the magnetized bead followed this rotary motion and as a result the linking number of the attached dsDNA changed. Thus, positive or negative coils were applied to or removed from the dsDNA. A rotation curve was generated by plotting the end-to-end length of the DNA molecule against the number of applied magnet rotations (Fig 1C). At any given magnet configuration, the overall linking number was conserved as the DNA was topologically constrained (i.e. not nicked).

### Flow cells

The bottom of the flow cell was a glass coverslip (24x60mm, Merzel Gläser) coated with 100,000 MW polystyrene (Sigma-Aldrich, 0.5% w/v polystyrene dissolved in toluene and spread onto a clean coverslip). Polystyrene beads in deionized MilliQ water were spread over the surface after which it was blown dry with a nitrogen gun. A spacer cut from a double layer of parafilm was placed on the coated side of this coverslip. On top, a clean, uncoated coverslip with two holes for inlet and outlet was placed. Consecutively, the parafilm was melted by heating the flow cell up to 85°C. Anti-digoxigenin antibodies (Roche Diagnostics) dissolved at 100 mg/ml in phosphate-buffered saline (PBS, Sigma-Aldrich) were incubated overnight at 4°C. Lastly, the flow cells were passivated by flushing in Bovine Serum Albumin (BSA) at a concentration of 10mg/ml (New England Biolabs) for at least 1 hour. Buffer exchanges occurred by removing the old buffer at the outlet by a syringe pump (Harvard Apparatus 11 Plus) while simultaneously pipetting new buffer into the inlet.

### DNA tethers

DNA molecules of 10kb length with different GC content were made by developing PCRs on genomic DNA from organisms with a GC content that was high (Streptomyces Clavuligerus DNA (DSMZ)), average (Unmethylated *c*l857 *Sam7* Lambda DNA (promega)) or low (Bacillus Megaterium QM B1551 DNA; a kind gift from the Beaumont lab) (S1 Table). The final GC content of the 6 different DNA constructs were 77%, 58%, 42% (asymmetric distribution of GC along the molecule), 42% (symmetric distribution of GC along the molecule), 39% and 38%. The GC content along the molecule is depicted in S1A Fig with a moving-average window of 25 and 250 bp. The difference between the two molecules that both contain 42% GC is the distribution of AT and GC along the molecule: The 250bp moving-average window of the 42% GC (symmetric distribution) shows a relative homogeneous distribution of AT and GC along the molecule (lower left panel in S1A Fig). The 250bp moving-average of the 42% GC (asymmetric distribution) shows a much larger variation of above 60% at the start of the molecule to around 30% from ~2500bp to 4000 bp along the molecule, followed by a varying GC content from 4–10 kbp (upper right panel of S1A Fig). The complete sequences and PCR and primer information of the tweezer constructs are described in S1 Table and S1 File. The PCR fragments were cloned in pCR-XL-topo using the TOPO XL PCR cloning kit (Life Technologies) and partially sequence verified (~1kb from each side, Macrogen). The PCR fragments were then isolated out of pCR-XL-topo using restriction enzymes (S1 Table) and gel purified (Wizard® SV Gel and PCR Clean-Up System, Promega). At one end of each DNA molecule, a handle (~600bp) with a number of biotin-labelled nucleotides was ligated. At the other end, a handle (~600bp) labelled with a number of digoxigenin nucleotides was ligated. The Biotin and Digoxigenin labeled handles were prepared by PCR on a pbluescriptIISK+ template (Stratagene) with a taq polymerase (GoTaq, Promega) and the addition of Biotin-16-dUTP (Roche), or Digoxigenin-11-dUTP (Roche) to the nucleotide mixture respectively. The forward primer was: GACCGAGATAGGGTTGAGTG, and reverse primer: CAGGGTCGGAACAGGAGAGC. The biotin-handle was digested with XhoI or ApaI (NEB) resulting in ~600 bp fragments. The Digoxigenin-handle was digested with NotI (NEB) resulting in ~600 bp fragments. The handles were purified (Wizard® SV Gel and PCR Clean-Up System, Promega), before ligation reactions. Ligation reactions were done overnight at 16°C, using T4 DNA ligase (Promega). The final construct was gel purified to remove unligated DNA handles. 10μl of streptavidin-coated beads (2.8μm in diameter, Dynabeads® M-270 Streptavidin) were washed 3 times in PBS, and finally resuspended in 10μl PBS containing 1ng of DNA and placed on ice for 20 minutes. After washing the flow cell with 0.8ml PBS, the DNA/bead mixture was incubated for 30 minutes, before the unbound beads were flushed out with 1ml PBS. All data were acquired in PBS buffer (10 mM phosphate buffer, 2.7 mM potassium chloride and 137 mM sodium chloride, pH 7.4, at 25°C).

### Rotation curves

Rotation curves were acquired from + 60 turns (+6% supercoiling density) to– 60 turns (-6% supercoiling density) at intervals of 1 turn. To allow the bead and molecule to relax to the new end-to-end length, a minimum waiting time of 3 seconds was applied after each magnet rotation (where we note that the 3 s relaxation time is at least two orders of magnitude larger than the estimated relaxation time due to the viscous drag of the bead). The average extension *Z* and its standard deviation (*std*) of each DNA molecule for each measurement condition was determined by averaging the position in 1024 time frames (at a video rate of 100Hz) and used as a single data point of a rotation curve. Rotation curves were made at seven different magnet heights. To minimize the effect of variation in magnetic content of the superparamagnetic beads, the rotation curves of several molecules were averaged, excluding those molecules of which the estimated applied force deviated by more than one standard deviation of the forces of all molecules. Uncertainty in height determination caused by variation in bead-attachment points, was corrected for by aligning the traces in *Z* based on the expected contour length. Since we were interested in the difference in extension between positive and negative supercoiling densities, this height alignment has no effect on the final results. For ease of comparison with other work, the data is shown as function of applied supercoiling density *σ* (i.e., 1 turn is a change in *σ* of 0.0010), instead of presenting the extension as a function of externally applied turns. The seven applied stretching forces were 0.2, 0.3, 0.5, 0.6, 0.8, 1.0, 1.2 pN, and the number of molecules used to generate the average curve was N = 18 for 77% GC, N = 21 for 58% GC, N = 9 for 42% (asymmetric spread of GC along the molecule), N = 19 for 42% GC (symmetric spread of GC along the molecule), N = 32 for 39% GC, N = 7 for 38% GC. The total number of molecules evaluated thus was N = 106.

### DNA topology

The topological state of a rotationally constrained DNA molecule is characterized with a linking number *Lk* = *Tw* + *Wr* [20,21]. Here, *Tw* represents twist, the number of times one DNA backbone makes a full turn around the DNA axis. *Wr* represents writhe, the number of plectonemic loops formed. Depending on the conditions, twist and writhe are interchangeable. The total linking number *L*_*k*_ remains constant, unless externally applied rotations change the *L*_*k*_ by *ΔL*_*k*_ (see Fig 1B). The supercoiling density *σ* is the amount of twist and writhe in a molecule relative to the natural helicity of B-DNA. When the linking number of a molecule is equal to the natural helicity of B-DNA (*L*_*k*,*0*_ = 10.45 bp per helical turn), *σ* is defined to be zero. Thus, *σ = (L*_*k*_ *-L*_*k*,*0*_ *) / L*_*k*,*0*_ *= ∆L*_*k*_ */ L*_*k*,*0*_.

In our force regime (up to 1.2pN), a few positive turns will result in slight overwinding twist in the DNA backbones. The energy associated with under/overwinding DNA is given by [22] the twisting energy $E_{twist}\;=k_{B}T\frac{C}{2l_{0}}\left(2\pi \cdot L_{k,twist}\right)$, where *C* is the DNA’s torsional modulus (≈86 nm [22]), *l*_0_ is the contour length of the DNA (3.4μm in this study), and *L*_*k*,*twist*_ is the number of turns absorbed by twisting the DNA.

After a certain amount of turns has been applied to the DNA, additional turns will not further increase the twist in the DNA backbone, but instead lead to the formation of plectonemes in the DNA. This transition point is called the buckling point. To form plectonemes with a radius *R*, the end-to-end length of the DNA is shortened, against the applied stretching force. Due to the stiffness of the DNA, it also costs energy to bend the DNA into a plectoneme. The total energy for plectoneme formation thus is given by [23]: $E_{plect}\;=\left(2\pi RF+2\pi R\frac{1}{2}\frac{B}{R^{2}}\right)\cdot L_{k,writhe}$, where *R* is the radius of the plectoneme loop, *F* is the applied stretching force, *B* is the DNA bending constant, and *L*_k,writhe_ is the number of formed plectonemes. The size of the plectoneme is dependent of the applied stretching force and bending constant [23], $R=\sqrt{B/2F}$. At a force of about 0.5pN, a plectonemic loop involves ~150 bp DNA, resulting in ~50nm decrease in the DNA extension. Similarly, at forces below 0.5pN a few negative turns will underwind the DNA helix, and extra turns beyond the buckling point will decrease the length by the formation of negative plectonemes.

In this study we were especially interested in the regime of negative supercoiling and forces between 0.5 and 1pN. In this regime there is a co-existence between melted and plectonemic DNA [8,12,24], as depicted in Fig 1D. The energy to melt DNA can be defined as [8] *E*_*den*_ = *α* ⋅ *L*_*k*,*den*_, where *L*_*k*, *den*_ is the number of turns relaxed by partial DNA denaturation and *α* is the average denaturation energy for a single turn. More specifically, *α* is the number of melted base pairs to denature one turn times the melting energy of a single base pair.

## Results

**Fig 2:**
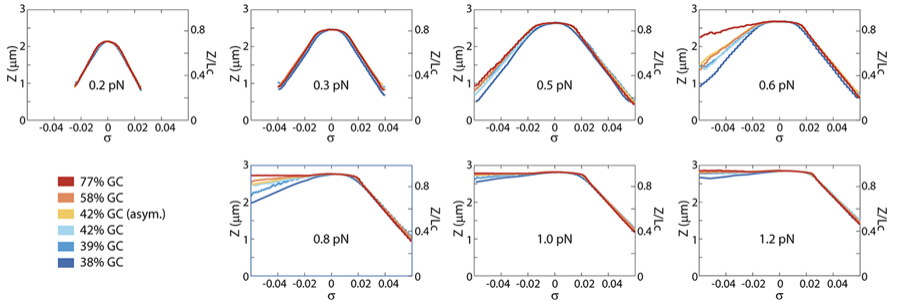
DNA extension as a function of supercoiling density. For six DNA sequences, the DNA extension *Z* is measured as function of the applied supercoiling density at varying forces between 0.2 and 1.2 pN. The shown curves are averages of a large number of individual molecules (see Materials and Methods). At 0.3pN and below all rotation curves are symmetric and applied supercoiling is absorbed into plectoneme formation. Increasing the applied stretching force results in less symmetric rotation curves. At intermediate forces of 0.6 and 0.8pN the sequence-dependent effect of competition between DNA melting and plectoneme formation is most pronounced. At 1.2pN all sequences show an asymmetric rotation curve: applied negative supercoiling is absorbed into DNA melting, and applied positive supercoiling is absorbed into plectonemes. For ease of comparison with other work, on the right axis the extension is presented relative to the contour length of the molecule (3.33μm). The noise in the extension (*std* of *Z*) of the molecules for these curves are shown in S2 Fig.

For six different DNA sequences with a varying GC content from 38% up to 77% (see Materials and Methods), we measured the DNA extension *Z* for positive and negative supercoiling densities, |*σ*| ≤ 0.06. Fig 2 shows the results for forces between 0.2 and 1.2 pN. At positive *σ* beyond the buckling point, all curves show a linear length decrease due to plectoneme formation. The plectoneme sizes, examined at the same force, are found to be similar for all different sequences, since the slopes (compaction per formed plectoneme) are similar. At negative *σ* and forces below 0.5pN, all the slopes are again similar. Above 0.5 pN, however, the extension for negative *σ* becomes larger than the extension for similar positive *σ*. This length increase is due to partial melting of the DNA [23]. The length increase is observed to be different for all sequences: Systematically, we observe that the higher GC-content DNA molecules exhibit a larger DNA extension (see e.g. the panels for 0.6 and 0.8pN in Fig 2), thus indicating a larger fraction of melted DNA. This counterintuitive finding (as one would expect AT-rich DNA to melt easier) is a key result of the current work.

**Fig 3:**
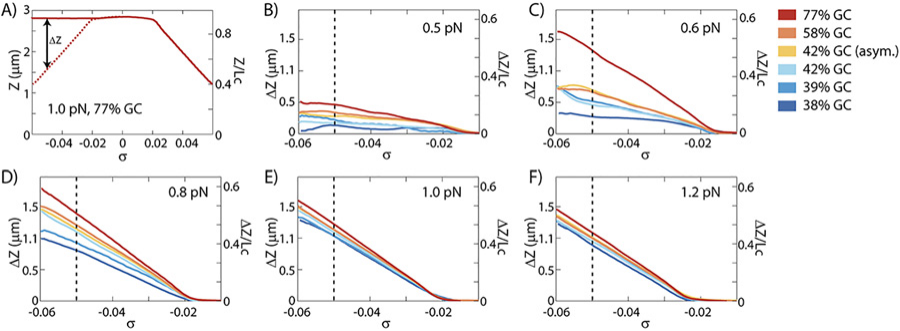
Length increase due to melting. The length increase due to melting, *ΔZ* is calculated from the extension at negative supercoiling density minus the extension at similar positive supercoiling density, *ΔZ* = 〈*Z*(−|*σ*|)〉 − 〈*Z*(+|*σ*|)〉. (A) Illustration of *ΔZ* with the data of the 77%GC construct at 1.0pN. (B-F) *ΔZ* as function of supercoiling density *σ* for forces between 0.5pN and 1.2pN. A positive *ΔZ* occurs when applied negative supercoiling induces melting where similar positive supercoiling induces plectonemes. At forces below 0.5pN, *ΔZ* is zero and no melting occurs. An increase in GC content results in a larger amount of melting and a smaller amount of plectoneme formation at the same force. The dotted lines at *σ* = -0.05 indicate the values of *ΔZ* used for Fig 4A in which the force dependence of *ΔZ* is shown. On the right axis the extension is presented relative to the contour length of the molecule (3.33μm). The difference in noise between positive and negative supercoiling (*∆std*) are shown in S3 Fig.

The length increase is shown in greater detail in Fig 3. In the force regime between 0.5 and 1.2pN, plectonemes and melted DNA co-exist. To separate the length increase due to melted DNA from the length decrease due to plectonemes, the extension at a certain positive supercoiling density (i.e. the length of DNA with plectonemes) is subtracted from the extension at equal negative supercoiling density (where DNA exhibits both plectonemes and melted DNA). Thus, the length increase due to melting is *ΔZ* = 〈*Z*(−|*σ*|)〉 − 〈*Z*(+|*σ*|)〉. Significant differences in *ΔZ* are observed for the different DNA sequences. Consistent with the rotation curves shown in Fig 2, we observe that a higher GC content leads to a larger fraction of melted DNA.

**Fig 4:**
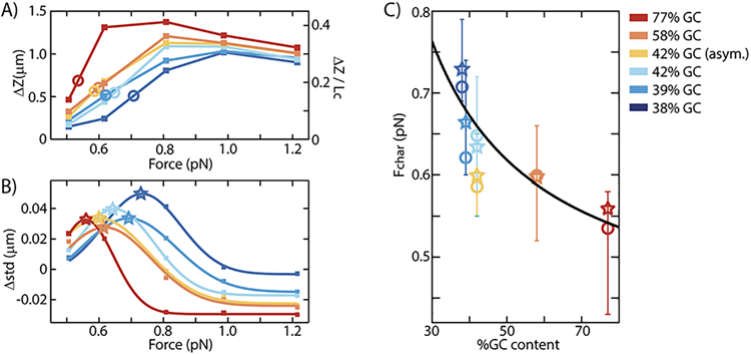
Difference in extension and noise at *σ* = ±0.05. (A) The difference in extension *ΔZ* = 〈*Z*(*σ* = −0.05)〉 − 〈*Z*(*σ* = +0.05)〉, as a function of applied stretching force. An increase in GC content results in a larger *ΔZ*, indicating more melting, which occurs at a lower applied stretching force. To determine the characteristic melting force *F*_*char*_, the force at half of the maximum *ΔZ* value per sequence is determined (open circles). The extension *Z* as function of force and *ΔZ* at *σ* = ± 0.02, 0.03, 0.04, 0.05 are shown in S4A Fig. On the right axis the extension is presented relative to the contour length of the molecule (3.33μm). (B) To quantify the noise on the signal in *Z*, the standard deviation in *Z* was measured for each molecule at each force and supercoiling density individually. For all molecules with the same sequence, the standard deviations under the same condition are averaged. This figure shows the force dependence of the difference in standard deviation at *σ* ± 0.05, Δ*std* = 〈*std*(*σ* = −0.05)〉 − 〈*std*(*σ* = +0.05)〉. A large standard deviation in *Z* at negative *σ* compared to positive *σ* in the force regime around 0.6–0.8 pN is indicative of the denaturation transition. To find the force at which the maximum in *∆std* occurs, a Gaussian fit to *∆std* has been made. The maximum in *∆std* (marked by stars) occurs at lower forces for GC-rich sequences, in agreement with the increase in *ΔZ* at lower forces for GC-rich sequences as shown in Fig A). More data on the *std* and *∆std* at different supercoiling densities are shown in S4B Fig. (C) The characteristic force *F*_*char*_ as function of the GC content. The open circles are the *F*_*char*_ values as determined from the *ΔZ* data. The stars are the *F*_*char*_ values as determined from the *Δstd* data for |*σ*| = 0.04, and 0.05. Similar values for *F*_*char*_ are obtained from *ΔZ* and *Δstd*. The black solid line is a phenomenological fit of *F*_*char*_ = (11/(%GC) + 0.4) pN. The transition from plectonemic to melted DNA occurs at lower applied stretching force for GC-rich sequences.

To map the effect of stretching force on the fraction of melted DNA, the length increase due to melting is determined for every measured force at a fixed supercoiling density. *ΔZ* as a function of force is shown in Fig 4A for |*σ*| = 0.05 (and in S4A Fig for |*σ*| = 0.02, 0.03, 0.04, and 0.05). With increasing force, the DNA molecules for all sequences show a strong initial increase in *ΔZ* up to a certain maximum, after which a weak decrease occurs. Sequences with larger GC content have an overall larger increase in *ΔZ*, which occurs at a lower applied stretching force. The critical force that needs to be applied in order to induce the melting of the double helix is thus lower for sequences with high GC content, which, as notes before, is a counterintuitive result.

We also determined the standard deviation (*std*) in the extension for each data point as shown in S2 Fig. As expected, relaxed B-DNA has a larger *std* for lower forces, due to thermal fluctuations. When applying positive rotations, the *std* remains similar up to the buckling point. At the buckling point a steep increase in the noise occurs due to plectoneme formation and the noise level in the plectoneme regime remains about 20-30nm higher than that of relaxed DNA. At negative *σ* below 0.5pN, the noise level is comparable to positive supercoiling. At very large forces, when only B-DNA and melted DNA are present, the noise level is comparable to relaxed DNA (see data at 1.2pN). In the transition regime (~0.5–1.0pN), a higher *std* is observed as thermal fluctuations may induce transitions between plectonemic and melted DNA. To compare the noise in *Z* at the transition region at negative supercoiling with the noise of the plectonemic region at similar positive supercoiling, *Δstd* is calculated, thus *Δstd* = 〈*std*(−|*σ*|)〉 − 〈*std*(+|*σ*|)〉 (S3 Fig). Fig 4B shows the force dependence of *Δstd* for |*σ|* = ±0.05 (data for multiple *σ* are given in S4B and S4C Fig). A peak in *Δstd* signals the transition from plectonemic DNA to melted DNA. Negative *Δstd* values at large forces indicate that the transition is completed. Again a sequence dependence effect occurs: the noise peaks indicate that GC-rich sequences exhibit a transition from plectonemic DNA to melted DNA at lower force than sequences with a lower GC content. Similar plots at different supercoiling densities show the same trend (S4B and S4C Fig).

## Discussion

The metabolism of DNA in all organisms relies on the balance between hybridized dsDNA and local de-hybridized regions. In contrast with predictions based on the melting temperatures of DNA [12–14], our results show that an increasing percentage of GC base pairs in a long DNA molecule *lowers* the stretching force necessary to locally dehybridize the DNA backbones in the presence of negative supercoiling densities. We quantified the GC content dependence of the DNA melting by determining a characteristic melting force, *F*_*char*_, using both the length increase due to melting as well as the increase in noise of the DNA extension at the transition force. Similar to Ref. [12], we define the characteristic melting force *F*_*char*_ as the force at which *ΔZ* is half the maximum length increase due to melting. *ΔZ* as function of force is shown in Fig 4A for |*σ*| = -0.05 (and S4A Fig for |*σ*| = 0.02, 0.03, 0.04. and 0.05), with *F*_*char*_ marked by the open circles. A more direct measurement of *F*_*char*_ is given by the noise level in *Z*, similar to Ref. [8], where we define *F*_*char*_ as the force at which the maximum in *Δstd* occurs. Although the maximum value of *Δstd* is dependent on the applied supercoiling |*σ*|, the force at which the maximum occurs is not (see S4C Fig). To find *F*_*char*_ from data such as in Fig 4B and S4B and S4C Fig, Gaussian fits to *Δstd* are made for each sequence. The results for |*σ*| = ± 0.05 are shown in Fig 4B, where the peaks of the Gaussian fits are marked by open stars.

Fig 4C thus is the key result of our data, showing a strong decrease for the characteristic melting force with increasing GC content of the DNA. The circles result from analyzing the length and the stars result from analyzing the standard deviation in the length of 106 individual molecules at 7 different forces (from 0.2 up to 1.2pN) and supercoiling densities from -6% up to 6%. Although independently determined, *F*_*char*_ obtained from the extension due to melting (Fig 4C, circles) is similar to *F*_*char*_ determined by the increase in noise (Fig 4C, stars). Both types of data show the unexpected result that an increase in GC content leads to a decrease in the characteristic melting force.

To understand this counterintuitive result, it is important to realize that the application of negative supercoiling densities of a few percent will only melt a few percent of the base pairs within the long DNA (S5 Fig). Thus even in the DNA with the highest GC content of 77% GC, studied at a supercoiling density of -6%, it is not necessarily the GC bases that melt. Given the smaller denaturation energy of AT base pairs compared to GC base pairs (ΔG ≈ 4.7 and ≈ 7.1 kJ/mole, respectively [13]), the denaturation bubbles will localize in areas with a high local density of AT base pairs, independent of the average GC-content. As a consequence, the sequence dependence of the characteristic melting force as shown in Fig 4C must arise from other factors that are involved in this transition from plectonemic to melted DNA (discussed further below). Note that these denaturation areas can be very small (~10 bp to relax 1 supercoil). Therefore, the reverse possibility, that the plectoneme formation would localize in the AT-rich part of the sequence without having to bend in the GC-rich part, will not hold, as an order of magnitude more bases are involved in plectoneme formation than in local melting. Quantitatively: the size of a plectoneme is ~115 (1pN) to ~150 (0.5pN) base pairs, as deduced from the slope of the rotation curves at positive supercoiling, which is a lot larger than the number of base-pairs that need to melt in order to absorb one negative turn, which is about 10, as the natural helicity of B-DNA is 10.45 bp per helical turn. Some negative supercoiling may perhaps be absorbed by a left-handed intertwining of the ssDNA strands of the melted DNA, but we expect that this is not a significant effect under the applied negative supercoiling of only a few percent, due to steric hindrance and electrostatic repulsion between the DNA backbones. At the characteristic force, the energy to melt ~10 bases thus equals the energy to bend ~145 base pairs. More extreme negative supercoiling densities are discussed in [25,26].

The characteristic force for the transition is the force at which the energy to bend a certain length of DNA into a plectoneme is equal to the energy to denature enough base pairs to relax one turn. In Ref. [8], the characteristic melting force is calculated by setting the energy to form a plectoneme ($E_{plect}\;=\;\left(2\pi RF+2\pi R\frac{1}{2}\frac{B}{R^{2}}\right)\cdot L_{k,writhe}$,) equal to the energy to melt the DNA (*E*_*den*_ = *α* ⋅ *L*_*k*,*den*_) when absorbing the same number of rotations (Δ*L*_*k*,*writhe*_ = Δ*L*_*k*,*den*_), yielding

$$F_{char}=\frac{\alpha^{2}}{8\pi^{2}B}\;,$$ (1)

where, again, α is the average denaturation energy needed to relax a single turn, and *B* is the DNA bending modulus [8,22]. As deduced above, a significant number of AT base pairs will melt before the melting energy of GC base pairs start to play a role in α. The melting energy of 10 (mostly AT) base pairs is approximately constant and importantly, independent of the overall GC content since local areas with a high density of AT base pairs occur in all sequences. Therefore we can assume that *α* is approximately independent of the average GC content of the molecules. Thus, as long as the supercoiling density is significantly lower than the percentage of AT bases, α does not strongly depend on sequence and Eq [1] becomes:

$$F_{char}\propto \frac{1}{B}\;.$$ (2)

This suggests that the sequence dependence of *F*_*char*_ in Fig 4C arises from a sequence dependence of the bending energy of the plectonemes. This would indicate that the bending energy associated with DNA plectonemes increases with increasing GC content. Previous reports have yielded contrasting results for this sequence dependence [27–30]. The physical picture that arises is that a stiffer DNA molecule (with more GC) will induce the AT-rich regions to melt earlier. *B* not only influences the characteristic melting force, but also the previously described buckling point ($\Gamma_{b}=\sqrt{2BF}$) and the plectoneme size ($R=\sqrt{\frac{B}{2F}}$). Based on our results for *F*_*char*_ we estimate the sequence-dependent effect of *B* on the buckling point and plectoneme size to be close to our measurement precision, and we therefore cannot use these as independent controls. Next to an increased bending energy for GC-rich sequences, an alternative explanation of the observed effect that GC-rich sequences melt at lower applied stretching forces than AT-rich sequences, could be an increase in torsional stiffness with increasing GC content, which will increase the buildup of torque, lower the number of turns at which plectoneme formation starts and thus lower the number of applied turns at which the DNA starts to melt. In other words, an increase in the torsional modulus *C* with increasing GC content can also contribute to a decrease of the characteristic force *F*_*char*_. Our current experiments cannot well distinguish whether an increase in bending stiffness or torsional stiffness, or a combination of both, underlies the observed effect. The important conclusion that GC-rich sequences melt earlier than AT-rich sequences holds either way, however.

In conclusion, we have found a sequence-dependent effect on the melting of DNA under biologically relevant supercoiling densities and stretching forces. For DNA sequences with a GC content varying between 38–77%, we determined the characteristic force at which the transition from plectonemic to melted DNA occurs (*F*_*char*_). Although AT-rich sequences have a lower melting energy than GC-rich sequences, the latter DNA molecules were found to exhibit a lower *F*_*char*_: *F*_*char*_~0.56pN for 77% GC and ~0.73pN for 38% GC. This unexpected effect can be explained with a DNA-construct-independent melting energy (as all sequences contain at least a few percent AT) in combination with a DNA-dependent stiffness. Due to the larger bending/torsional energy of GC-rich DNA, the transition from plectonemic to melted DNA occurs at lower stretching forces. DNA denaturation plays an important role in many important processes in the cell and most cells actively impose an overall negative DNA supercoiling density of a few percent. Our results show that it is not only the presence of AT base pairs that regulate local openings of the DNA. The stiffness of adjacent GC-rich regions additionally enhances the opening of the DNA to provide accessibility for important protein-DNA interactions.

## Supporting Information

S1 Fig GC content and distribution for the six DNA sequences. (A) The GC content along the molecule is depicted for each sequence, as indicated by the colors. The colored (black) traces are the data for a moving average with a window of 25 bp (250bp). The complete sequences of the used constructs are given in the S1 File. (B) The length distribution of GC runs, i.e. runs of adjacent stretches of bases without any A or T bases, for each sequence. (C) The length distribution of AT runs for each sequence.
(TIF)

S2 Fig Supercoiling density dependence of the noise on Z. The standard deviation (*std*) in *Z* is determined from 1024 frames per individual molecule. Each panel shows the supercoiling density dependence of the *std* as deduced at various forces. Around *σ* = 0, the noise in *Z* is minimal. At positive *σ*, the noise increases at the buckling point where the transition to plectoneme formation takes place. At negative *σ*, especially around 0.6 and 0.8pN, the *std* increases significantly, indication the transition from plectoneme formation to melting. A clear sequence-dependent effect occurs: At 0.8pN stretching force, the sequence with the low GC content has a large *std*, indicating the transition from plectoneme to melted DNA, whereas the low *std* for GC-rich sequences indicates that the transition already occurred.
(TIF)

S3 Fig Difference in extension noise between DNA at positive and negative supercoiling densities. By subtracting the noise in *Z* (*std*) at positive supercoiling from the *std* at comparable negative supercoiling, Δ*std*(−*σ*) ≡ 〈*std*(+*σ*)〉 − 〈*std*(−*σ*)〉, the effect of melting on the stability of the extension of the molecule becomes clear. Between 0.5 and 0.8 pN the *∆std* values are increased compared to the values at positive *σ* due to the transition from plectoneme to melted DNA. The negative values for *∆std* at 1.0 and 1.2pN indicate that melted DNA is more stable in *Z* than plectonemic DNA.
(TIF)

S4 Fig Extended experimental data sets for Z, ∆Z, std and ∆std. (A) The extension *Z* as a function of force for both positive (left panels) and negative (middle panels) *σ*. The difference in *Z* between comparable supercoiling densities, *∆Z*, as function of applied stretching force is shown in the right panels. From top to bottom, the applied supercoiling densities are: |*σ*| = 0.02, 0.03, 0.04, 0.05. Increasing *σ* shows a more pronounced, but similar effect: Sequences containing high GC content melt at lower forces than sequences with lower GC content. (B) The force dependence of the standard deviation in *Z*, *std*, is shown for a number of supercoiling densities (± 0.02, ±0.03, ±0.04 and ±0.05). At positive *σ*, lower force results in a larger *std*, due to Brownian motion. At negative supercoiling the transition between plectoneme and melted DNA adds more noise to the extension signal. The difference in noise between positive (left panels) and negative (middle panels) supercoiling densities is shown in the right panels. Increasing |*σ*| shows a more pronounced, but similar effect: Sequences containing a high GC content exhibit the transition from plectonemes and melted DNA at lower forces than sequences with a lower GC content. (C) Data of the right panels in B), as ordered per sequence to show the effect of supercoiling density on *∆std*: increasing |*σ*| gives a more pronounced, but similar peak in the data for *σ* = -0.03, -0.04 or -0.05. At *σ* = -0.02 no significant melting occurs for most sequences.
(TIF)

S5 Fig Percentage of melted base pairs as function of applied negative supercoiling. The number of melted base pairs are calculated as follows: First, the length increase due to melting, *ΔZ*, is divided by the length increase due to a single plectoneme (slope of the length decrease at positive *σ*) to obtain the number of absorbed plectonemes. Second, the number of absorbed plectonemes is multiplied by 10.45, the number of base pairs in one helical turn. Since the buckling point occurs between *σ* = -0.03 and *σ* = 0, the changed helicity due to twist absorption is negligible. Third, the number of melted base pairs is divided by the total number of base pairs (10,007). The maximum fraction of melted base pairs is about 4% for *σ* = -0.06. As it should be, in the regime where a coexistence of only melted and B-DNA (1.2pN), the fraction of melted DNA shows a one-to-one relation with the applied supercoiling density beyond the buckling point.
(TIF)

S1 File Sequences of the used constructs. (PDF)

S1 Table PCR and primer information of the Tweezer Constructs. Sequence of the primers used to PCR the 10kb DNA fragments which were used to clone into pCR-XL-Topo vector. All PCR’s were performed using KOD Xtreme polymerase (MerckMillipore). To be able to make the 77% GC construct we first developed two PCR’s ~5kb, which were cloned into pSuperCos1 (stratagene), using restriction enzymes specified (partially sequence verified). From this plasmid the 10kb PCR was developed and cloned into pCR-XL-Topo vector.
(PDF)
