# Supplementary figures and images for: Counterintuitive DNA Sequence Dependence in Supercoiling-Induced DNA Melting
Source: PLoS One. 2015 Oct 29;10(10):e0141576. doi: 10.1371/journal.pone.0141576 (PMC4625975; doi:10.1371/journal.pone.0141576)

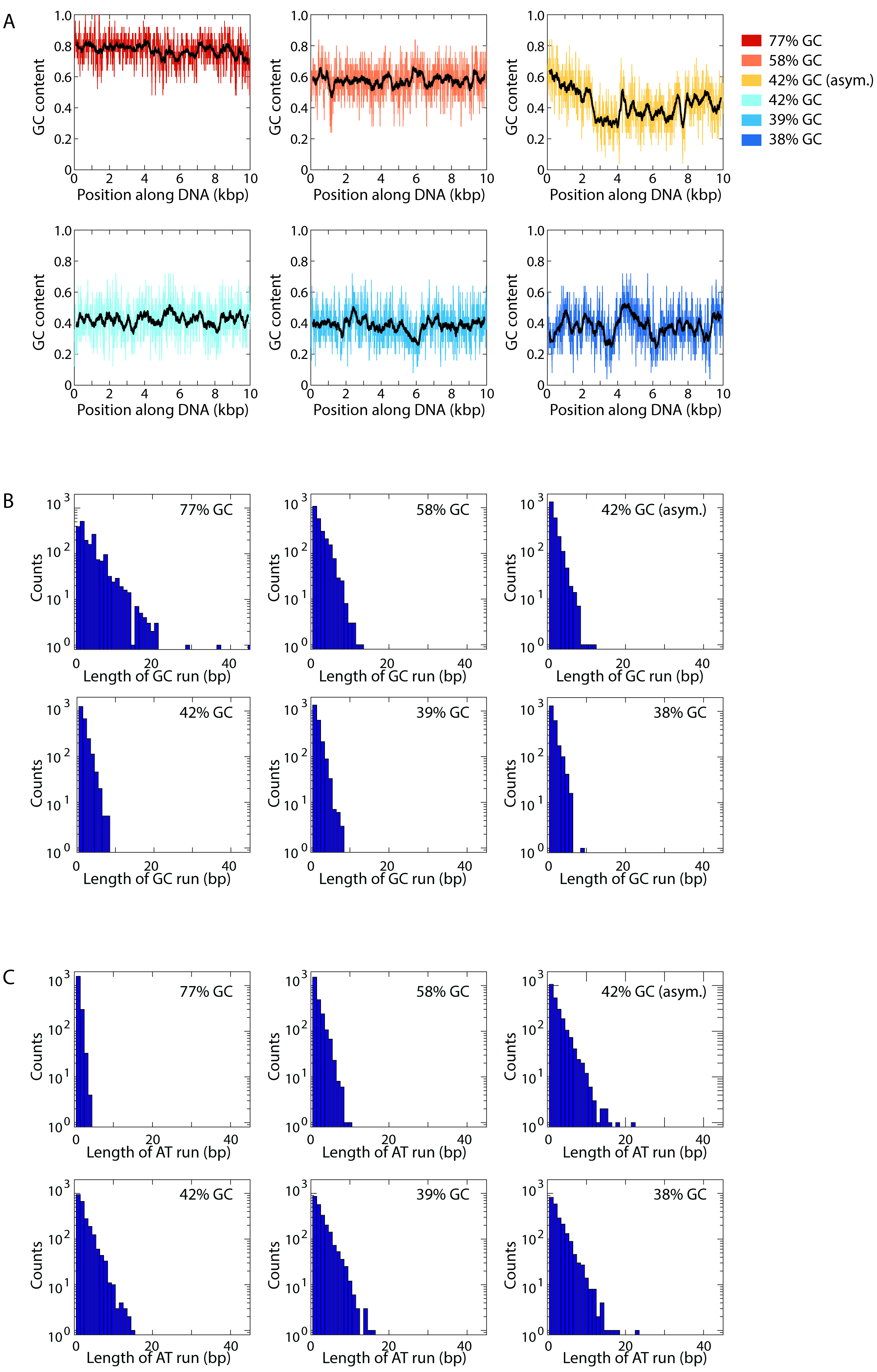

Supplement: S1 Fig — (A) The GC content along the molecule is depicted for each sequence, as indicated by the colors. The colored (black) traces are the data for a moving average with a window of 25 bp (250bp). The complete sequences of the used constructs are given in the S1 File. (B) The length distribution of GC runs, i.e. runs of adjacent stretches of bases without any A or T bases, for each sequence. (C) The length distribution of AT runs for each sequence. (TIF) [file pone.0141576.s001.tif]

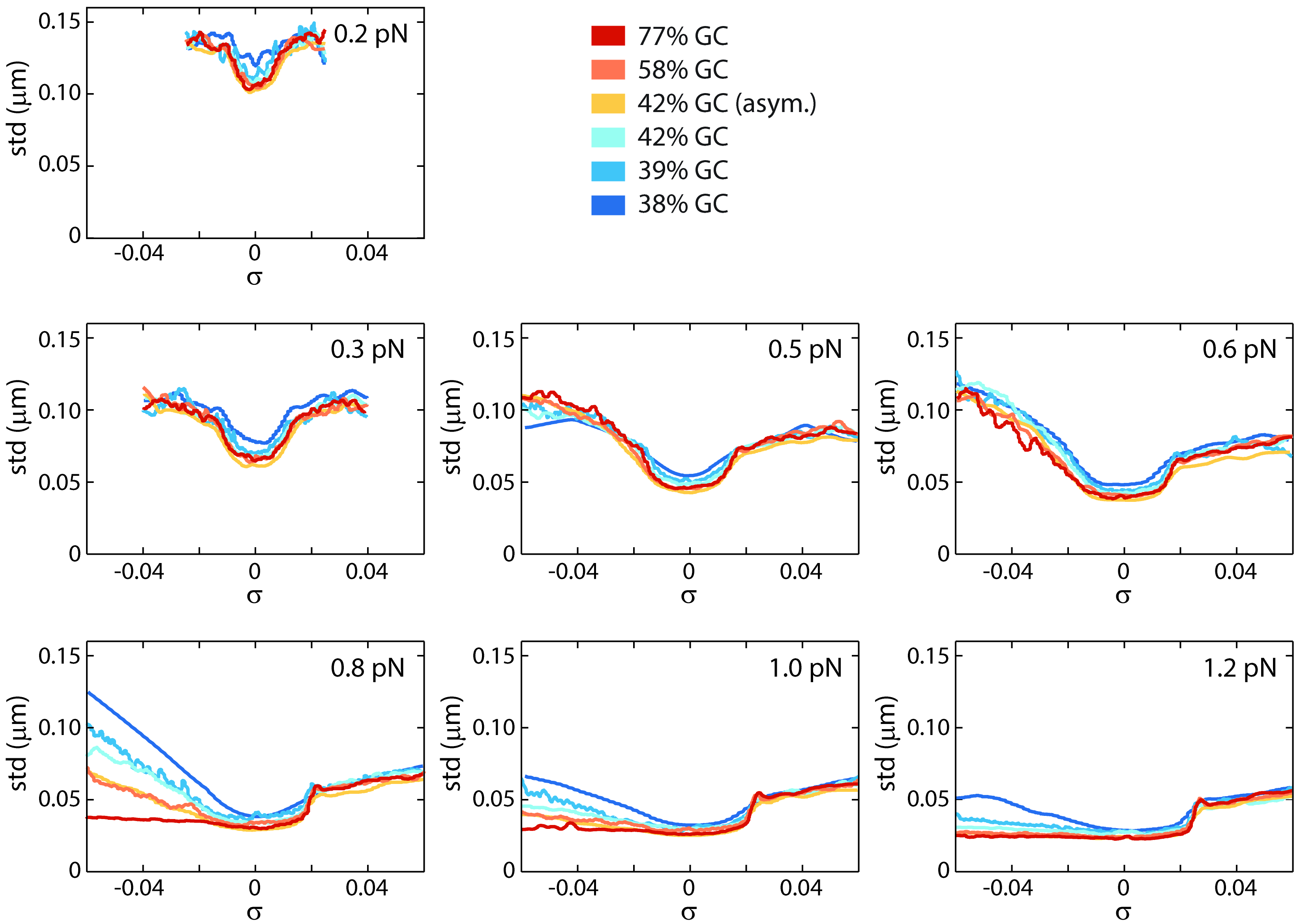

Supplement: S2 Fig — The standard deviation (std) in Z is determined from 1024 frames per individual molecule. Each panel shows the supercoiling density dependence of the std as deduced at various forces. Around σ = 0, the noise in Z is minimal. At positive σ, the noise increases at the buckling point where the transition to plectoneme formation takes place. At negative σ, especially around 0.6 and 0.8pN, the std increases significantly, indication the transition from plectoneme formation to melting. A clear sequence-dependent effect occurs: At 0.8pN stretching force, the sequence with the low GC content has a large std, indicating the transition from plectoneme to melted DNA, whereas the low std for GC-rich sequences indicates that the transition already occurred. (TIF) [file pone.0141576.s002.tif]

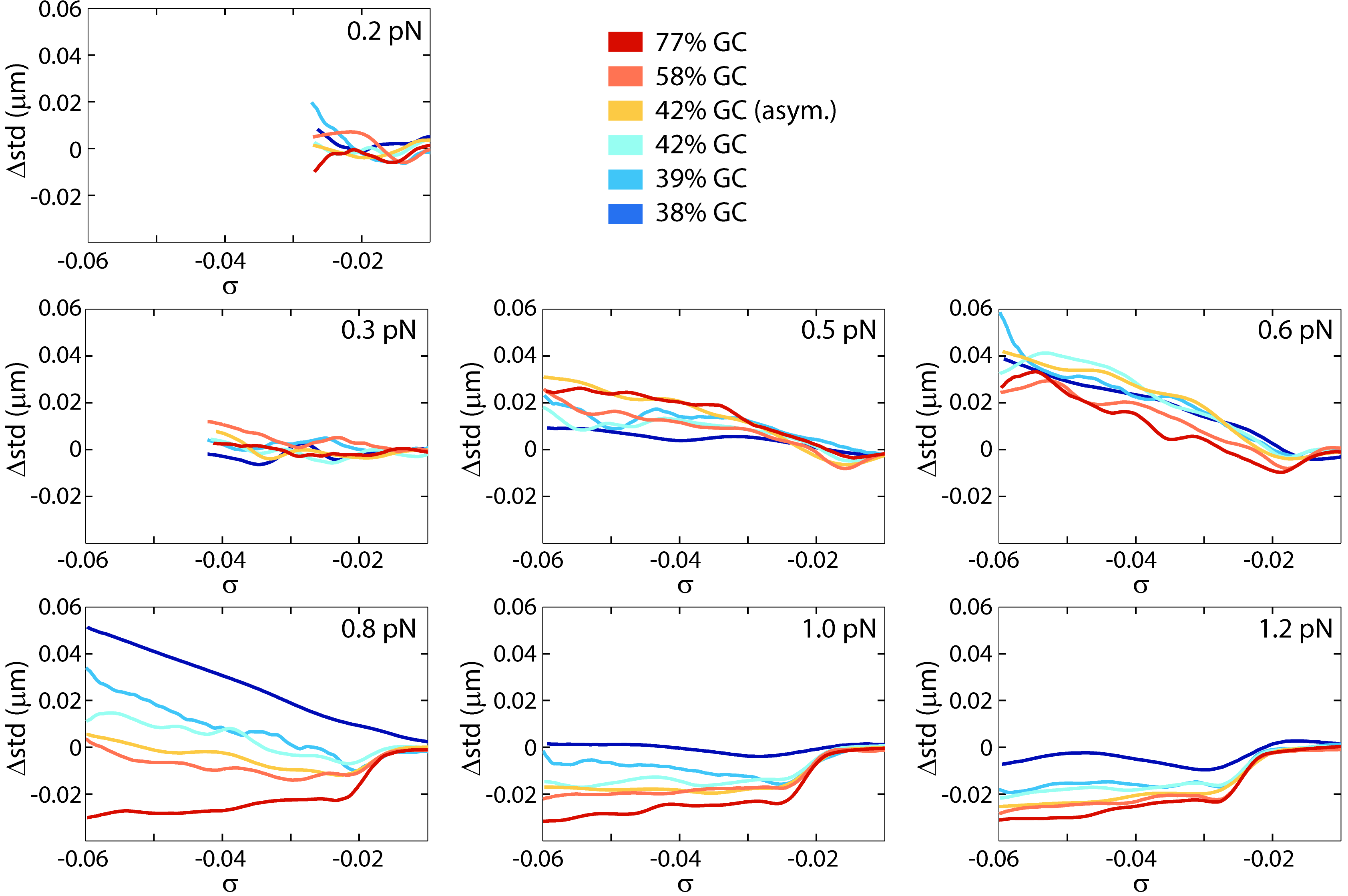

Supplement: S3 Fig — By subtracting the noise in Z (std) at positive supercoiling from the std at comparable negative supercoiling, Δstd(−σ) ≡ 〈std(+σ)〉 − 〈std(−σ)〉, the effect of melting on the stability of the extension of the molecule becomes clear. Between 0.5 and 0.8 pN the ∆std values are increased compared to the values at positive σ due to the transition from plectoneme to melted DNA. The negative values for ∆std at 1.0 and 1.2pN indicate that melted DNA is more stable in Z than plectonemic DNA. (TIF) [file pone.0141576.s003.tif]

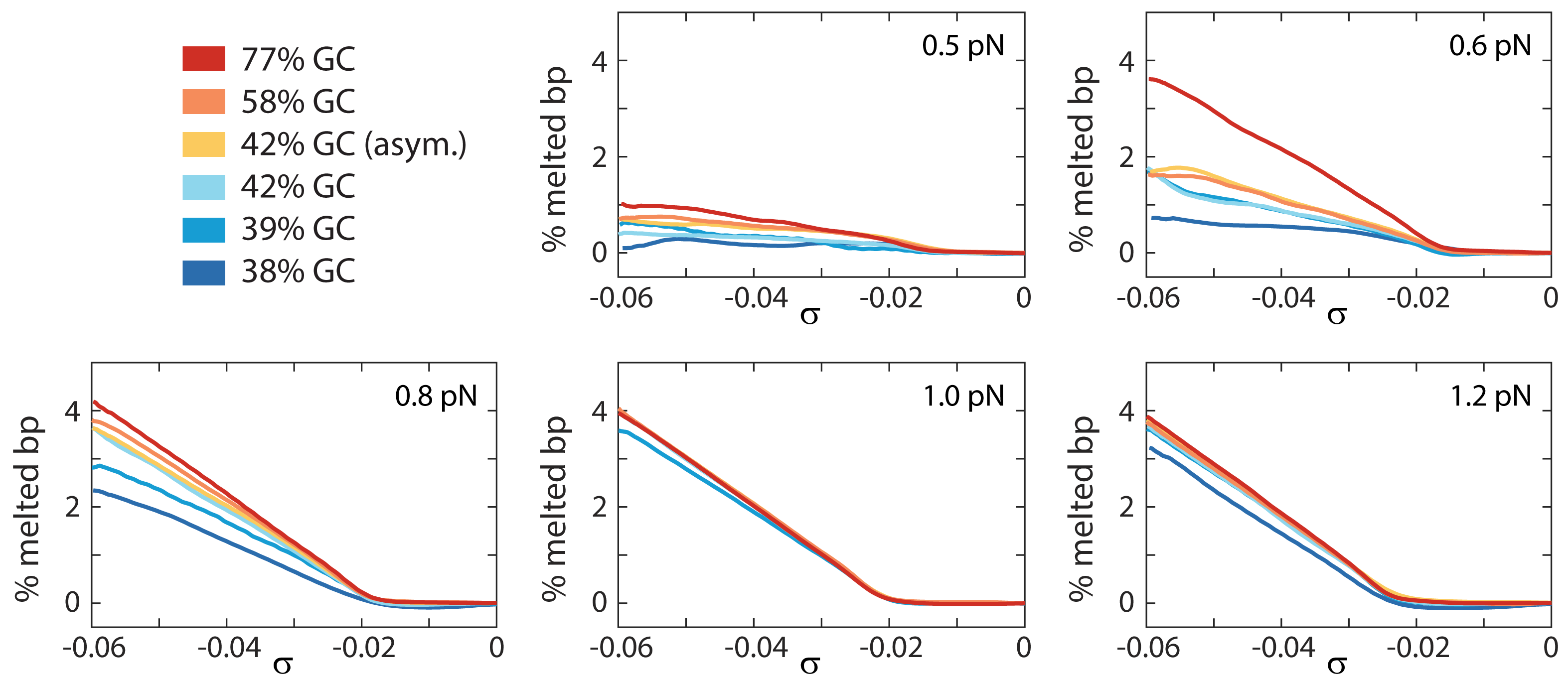

Supplement: S5 Fig — The number of melted base pairs are calculated as follows: First, the length increase due to melting, ΔZ, is divided by the length increase due to a single plectoneme (slope of the length decrease at positive σ) to obtain the number of absorbed plectonemes. Second, the number of absorbed plectonemes is multiplied by 10.45, the number of base pairs in one helical turn. Since the buckling point occurs between σ = -0.03 and σ = 0, the changed helicity due to twist absorption is negligible. Third, the number of melted base pairs is divided by the total number of base pairs (10,007). The maximum fraction of melted base pairs is about 4% for σ = -0.06. As it should be, in the regime where a coexistence of only melted and B-DNA (1.2pN), the fraction of melted DNA shows a one-to-one relation with the applied supercoiling density beyond the buckling point. (TIF) [file pone.0141576.s005.tif]
