# Supplementary material for: Counterintuitive DNA Sequence Dependence in Supercoiling-Induced DNA Melting
Source: PLoS One. 2015 Oct 29;10(10):e0141576. doi: 10.1371/journal.pone.0141576 (PMC4625975; doi:10.1371/journal.pone.0141576)
Supplement: S1 File — (PDF) [file pone.0141576.s006.pdf]

Construct 1, 77% GC content (excluding handles):

GGCCGCCCAGGCCCACCCCCTCGTGGACGAGGCCCTCGCCGTCCACGCCCTCCCCGGCCGCCCCGCGCGGACCGCCCGCTTCCTCGCCCTGCGCGCCGCCC
TGCTCTGCGGGGGCCGCCGTCTCGACGAGGCCCGGACCCTCGCCGACCAGGCCCTGGAGCGCTCACTCGCCGTACGGGCCCCCGTCGCCGAGGCGTACGC
CCGTTCCGTCCGCGCCCAGTTGCTCACCCACGCGGGCGACCACCCGGCGGCGCTGCGGGAGTCCGTCCGCGCCCGCACCGCCGCCCGGCGGCGCGCGGA
GACCCGCGACATCCAGCTCGTCCAGACCCTCCTCGGCGCCCATCTCCTCGGGGTGCACGACCGCGACGGCGAGGCCCGCGCCCTGCTGGAGGAGGCCAGG
GCGCTCGCCGACAGCACCGGTTCGCCCGCCCGGAAGGCATGGGCGCACACCGTCTCCGCGCGCTTCCACTACCGCGCCGGGCACTGGGACGAGGCCCTCG
ACGACCTCGACCTCGGACGCTCGCTGCCCGCCCCCTGGCAGCCCTCCCCGCCGTACGGGCTCGACGCCGTGATCCTCGTCGGCCGGGACCGCCGCGACGC
GGCCAGGGCCGCGCTCACCGCCGCCCGCGCCGCCGCGCCCGCCGGGACCGACCCCGGCTATCTCCCGCTCGCCGAGGCCCTGCTCGCCGAACGCGACGGC
GACCCGCACGGCGCGCTCGCCGCCCTCCGGGACGCGCTGTGCGAGGAGCCCCCCGGCCGCTGGAGCCACCGGGCCTTCTGGCTCCCCGACACCGTCAGGA
TCGCCCTCGCCCTCGGCGACCGGACGCTGGCGGCCACCGCAGTGACCCTCGCCGGGGCCCTGGCGGACACCGCGCCGGGCGAACGGGGCCCCCGCGCGC
TCGCCCAGCGCTGCCGGGGCCTCGCCGAGGGCGAACCCGGGGCGCTGCGCGCCGCCGCCGACCACTACCAGCGCCAGGGCATGCGGCTCTGTCTCGCCC
GCACCCAGGAGGACCTCGCCGCCGTGCTGGCCGCGCAGGGCGACACCGACGGGGCCCGCCGCGGCCTCAACCACGCCGTCGACCTCTACCAGCTTCTCGC
CGCCCACTGGTACACCGCCCGCGCCGACGCCCGGCTGCGTGCCCTCGGGGTCCGCCGCGGCCCCCGGGGCGCCCGGGGCCGCCCCCGCACCGGCTGGGA
CGCCCTCACCCCCACCGAACTGAAGGTCGCGCTCCTCGTCGCCGAAGGCCGCTCCAACCCCGAGGCCGCCGCCGAACTGCTGCTCTCCCCGCGCACCGTCC
AGACCCATGTCTCGCACATCCTCACCAAGCTCGGCGCCCGCACCCGTATCGACATCGGCAGGGAAGCGGGCCGCCGACGGCCGCTGAACGGACCGGCGG
CCCCGCCCCGCGGGGCCGCCCCGGCCCCCGCGCCCGCCACCGGAAAGGAGACCGACGACCGTGAAGGCCGTGACGACGGGGAGGGCCCGCGGCGACGA
CGGCGTCTGGACCGGCAGAGCCCCCGAGACACAGCGGTTGAGCCGGTACATCGCGGAACTGGCGAGCGGCCGGGGGCACTCCGTGCTGATCGAGGGCG
AACCCGGCATCGGGAGGACCGCGCTGCTCCACCGGGCCCGGCTTGAGGCCCGGCGGACCGGCCGCACCACCCTCGCCGCGACGGCCGAGGACGGGCAC
ACCCCCCTGCGCCCCGTCCTCGAAGCCCTCGCCCGGCCCGGACCCGTCCGCCCCGTGCTGCGCGCCCTCGCGGCCGGACTGCTCCGCGGCCCCCAGCCCCG
TATCCCCGCCACCGCCGCCGGGGAACCCGATCCCGCCCTCGCCGAACGGGTCCTCGACCTGATCCGCCGGGTCACCGACGAGGGACCGCTGGCCCTTCTCC
TCGACGACCTCCACCGCGCCGACCCCACCACCCTGCTGCTGTGGCAGCGGCTCGCCCGGCGCACCGCCCGGCTGCCCCTGCTGCTCGTCGCCACCGCACGC
CGCCTCCCCGGCCCCGGCGGCCCCGGTGGATTCGCCGGCCTCCGGCGCGACCTCCTCGACGCGGGAACCACCCTGCTCACCCTGGCCCCGCTCGCCGACGA
CGAGGCCCGCGCGCTCGTCGGCGCCCTCACCGGCGCCCCGCCCGGCCCCGAACTCCGCGAACGGATCGACCGGGCGGGCGGCAACCCCCACTGGATACG
GACGCTGCTCCCCCCCGACCGCACGGAACGGAACCCGGCCCCTCCGACCCCCCTGGCCCCTCCGACCCTTCCTAACCCTCCGGTTCCTCCGGCTCTCCCGGG
CCCCGCGGCCCGTCCGGCCGACCCGCCCACCGGCACCGGCTCAGGCTCCGGCGGCAGCTCCGGCAGCTCCGGCAGCTCCGGCAGCTCCGGCAGCTCCGGC
AGCGGCGGCTCCGGTCCCGCTGCCGCCACCGGCCTCGCCGCCGTCGCCGACGGTCTCGTCGACGGCGCGACCCGGGACACCCTGCGCACCGCCGCCCTCC
TCGGCCCCGCCTTCACCGCCGCCGAACTCGCCGCCGTCACCGGACGGCCACCGCTCGACCTGCTGGACCCCCTCGACGACGCCCTCGCCGCCGGAGTCCTC
GTCGACACCGGCGACCACCTCCGCTTCCGCCTCCCCGCGCTCGCCGAGGCCCTGCGCGCCACCGTCCCCGCCCCCGAGCGCGCCGAACACCACCGCGCCGC
CGCCCACGCCCTGGCCGCGGCGGGCGCGGACCTCGAACGCGTCGCCGGACAACTGCTCCCGATCGCCGGAACCCTCACCGCCGACCGGGACGGCTGGGC
CTTCGACTGGCTCGCGGGCGCCGCGCCCCACCTCGCCGAACAGGCCCCCGGCAGCGCCGTCGGCCTGCTGCGCCGCTGTCTGGCCCGGCTCCCCGCCGAC
GACCCCCGGCACGCCGCCCTCAAGGAACACCTCGCCCACGCCGCCCTGTTGCTGCGCGGCCCCGCGAGCACCGCGACCCTGCGCGGACTCCTGGACGAGA
CCACCGCACCCGAGGCCCGCGTCCAGCTCACCGCCGCGCTCGCCCACGGCCTGTACGTCCAGGGCCGCTGGGCCGACGCCCTCGCCGCCCTCGACCGGGC
CGAGCGCACCGGAGCCGCCGACGCCGCCTGGGCCCCGCTCTTCCAGGGGCTGCGCGCCACGATCCACCACGGCGCCGGTCACAGCGAGCGGTGCGCGGT
CCTCGCCCGGCAGGTCATCGCCGACGCCCGGTCCGGACGGCACCCCTTCGGCGAGGCATACGGGCGGCACGCCCTGTCCTGCGCCCTGCTGCGGGACCGG
CGCACCGAGGCGGCCCTCGCCGAGAACACCCTCGCCCTGCGCGCCGCCGCACGGGTGGACCGGACCGGCGCCCGCTGGCTCACCTGCACCCTCACCGACC
TCCGCGTCACCATGCTGCTCTACCGGGCGGTCATGCTCGGCCTGGTCGACCGGCCCGAGGAGGCGCGGTCCGCGCTCGACCGGGCCCGGGACGAGACGG
CCGGCCGGGCCACCGCCGATCAGCTCGGCGGCATCACCGTGACCGCCGCGCTCCTCGACTACACCACCGGCCGCTGGGACGACGCCCTCGCCCGGCTGGA
CCGGCAGCCCGACCCCGTCGACCCCTGGCTCCCCTCCCTCCGCCACAGCGTCGCCGCCCTCGTCCACGGCCACCGCGACCGGGGCGCCGACGCCCGGGCCC
ACTGCGAGGCCGCCGCCGGGCGGACCACCCCCTGCGGCTCGCACAGCAACCGCGGGGGCTATCCCCTCATGGCCCGCGCGCTGCTGGCGGAGCGGTCCG
GCCGCCCGCACGAGGCGCTGGCGGTCCTGCTCCCCACCCTCGACCCCGGCTACGCCCGCGACCTGGACCAGCGCTTCCAGTGGATGCCCGACATCGTGCG
GCTCGCCCTGCGCACCGGGGACGGGGCCACCGCGCGCGCCGCCGCCGTCATCAGCGCCGGGGAGGCCGACCGCGAACACCACCCGGCGCGGACCGCCG
CCGCCGCACGCTGCCGGGGGCTCGTGGACGCCGACCCCGGCCCGCTCGCCGAGGCCGTCGCCTACTACCGCGGGGTCGCCCGCCCGCTCGACCTCGGCCA
GGCCCTGGAGGACGGCGCGGCCGTCCGGGCCGCGCTCGGCGAACCGGCGGCGGCCAGGGAACTGCTGCAACAGGCCCTGGAACGGTACGCGCGGCTCG
GCGCGGCCTGGGACGCCCGGCGCGCCGTCGGGCGGCTGCGGGCCCTGGGCGTCCGTACGGGCCCCCGGGCCGCCCGGAACGGACGCCCGTCCACCGGC
TGGGCCGCCCTCACCCCCGCCGAGCTGAGAGTGGCCCACCGGGTGGCCCAGGGCCGCTCCAACCCCGAGATCGCGGCGGAGCTGTTCCTCTCGCCGCGCA
CCGTCCAGACCCATGTCTCCAGCATCCTCACCAAACTCGGCGCCCGCTCCCGTACCGAGGTGGCCCGCCAGGCCGCCGAACGCTCCTCCCAGCACCCCTGA
CCGACCGGCCCGGCCGGACCGCACGACCCCGCCCGGAGCGGTCGGCGCGTACGTCGCGAGGGGGGCGCGCGGCCGTGAACCCGCGCCCCCGTCACGCC
GTGCTCCGGAGGGAGAGTTCCATCTCCTGACCTGTGAGGATTCCGCATGGCACCGCACGATCCCCAGAGCCCGTACGGCCCCGCGCACCCGCCGACGGGC
GCGCCGGACACCGCCATCGGCGGCGGCGCCACGCCCACCGGCCCCAGGGCCCCCAGAGGTACGGCCCGTACCTCCTGTCGACTGACGGCGCACGAGGGG
GGCCGGGCCGACGGGTGAGCGGGAGACCGGTGCCGACGGCGACACGCCGACGGCCCTTTCGGCCCTTGTTGTTGTGCGGCACCTGTCCGCAGAATGGCC
CCGGGGGCGGGCCGGAGGATGGCTCCGGTGCGCCGGAGCACACGAATCGGGGGCCTGCGTGACTGAGCCGCACCGGGCGGAAGCCGGATCCCCGGGT
GTCCTCGGCTTCCTGGTCTTCGCCGCGCTGGCCGTGGGCGTCTGGTTCCTGATGAAGTCCCTGAACCGGCACATGGGCCGGATCAACTTCGAGGATTCCCC
GGCGCCGGAGAAGAGCACGGGCAACGGCACCGGTTCCGGTGCCGTTGCCGGAACCGCCGCGCGCCCGGCGGTGAAGAGCGGGGCCGGGGCCGCCGAG
GTCACCACGACCACGACCACGACCACGGCCGCGAAGGCGACGAAGACCACAAAAGCCGCGAAGGCCACGAAGAAGGTCTGACCCGGTCCGGGTCCGCTC
CGCGCGTGCGGGGCCGACCCGGACCGGACGCCCGGCCGGCCCGGAGGCGGTCACCCCGCCTCCGGGCCGGTCCGTGCGTTGTCCGTACCCCTGTACCTCC
GTGGCCCTCAGGCCGTCGGGCGGTCCACCGGGACGCCCATGACCTCCCGCGCGTGCCGGTTCGGCACCAGGCCGAGCTGCCACGCCTGCCAGCCGTCCTC
CAGCCGGACCCCGCGCTCCAGCACCACGCCCAGCGCCCCGGCGCAGTCTTCCAGCTTTCCGTCCCGTGCGGGGTGCTTCGCCAGGACCAGCCGGGACAGC
TCCTCCTGCGCGACGACCGTCCCCACCTCGACCCCGCCGGGCGACGCGTACGGCAGCAGCGTGCACCGCAGGAACCGGGCCCAGTCGCCGCCGCGCGCGT
CCTCGTACGAGGCGAAGAGCGCCACCGCCTCGTCACAGAGCGCCAGCGCCTGCTCGGTACGGTGGTTGCCCGCGTCGATCAGGGCGAGTTCCAGACAGG
TCCATGCCTCGCCATGGGCGACGCCGATCCGGCGGAAGTCATTGCGGCAGTCCACGAGGAGCTGACGGGCGAAACCGGAGTTGCGGAGGTTGCCCGTCC
GCGCGGCCCGCTGGTCCCGGGTGACCCGTCCCGAGTGATGACGGGCGCAGGCCAGCCCGTACACGTCGCGCATCCGGGAGAACATCGTGCGGGCCCGTT
CCAGCTCCCGTACCGCCCGGTCGAGATCGCCCTGCTCCTCCAGCGCCTGCCCCAGGTAGTACAGGGTCCATGCCTCACCGCGCACGTCCTCGTTCTCCCGAT

GGCCGGCCAGCGCCTCCCGCAGCCCGTCCACCGCCGCCGAGGGCTCGCCGTCCACCAGCCGGGCCCGCGCGAGCTGGGTCAGCGCCCACGCCTCACCGCG
TCCGTCGCCCGTCCGCCCGTACAGCTCCAGAGCGGTGCGCAGCTCCGTCTCGGCCTCCGGGACCGTGCCCATCCGCAGATACACCTGGCCGAGCTGGAAGT
GCGTCCACGCCTCGCCGTGCAGGGACTCGCCCTCGCGGTGCAGCTCCAGCGCGGTCGCCAGCAGGGACCGGGCCCGGGCGAGATCGGCCTGGTCCCGGC
GGACCGCCGCGAGCGCGTGCAGCGACCAGGCGCGGTCCTCCGCCTGCCCGGCCGGTGCCTGGAGCGCGACGGCCTCGGTCAGCCTCGCCGCGGCCTCCG
TGAGATTGCCCTGGTGGTGCAGGGTGATCCCGAGCGAGCAGAGCGCGAGCGCCGTCCCCGCGTCGTTGTGCTCGTCGCGGTAGCGGCCCACGACGGACG
ACAGCGTGGTGCGGGCCTTGTCCAGCTCGCCGAGCTGACGCGCCGCGATACCGGTGCGCCACTGCACCGACCGCTCCAGCGGGCCCGAGTCGCCCCCGCC
CTGGCCGACGGCCTGCGACAGCTCGCTGATCTCGCCGAGGCGGTAGAGGTCGCCCCGGAGCAGACAGTGATCGGCGAGCGCGCCCAGCAGCCGGAGCA
CCGTGCGCTGGTCCACGCCCTCCGCGTGGCGCAGCGCCGAGGTGATGAAGCTGGACTCCTCGTCGAGCCAGCGCAGCGCGGAGTCGAGGGACGGGAAG
CCGTGCGCGTCGAACCGGCCCGCGCGGGTGGACATCTTGCCGTCGACCATCCGGGTCACGGCCCCGGCGAGATCCGCGTAGTTCTGGATCAGCCGCTCCT
GTGCGGCGGTCCGCTCGGCGGCGTCCTCCTCGTCGCGGAGCCGGGCCCGCGCGAAGGCGCGGACGGCGTCGTGGAGGCGGTAGCGGCTGGACCGCACC
CGGTCGATCAGCCCGGCGTCGGCCAGCTCGGCCAGCAGCTTCCGCGCCTCCTGCCCGTCGGCCGCGAGCAGGGCCGCCGCGGCTGCGGCGCCGAGGGAC
GCCCGCCCCGCGAGGGCGAGCCGCCGCAGCAGCCGGCGGCCGGTCTCCGACTGGTCGCGGTAGCGGAGCCAGAGGGCGCGCTCGACGGGGTCGGAGCC
CGGCTGGGCGGCGAGTCCGTCGGCGAGCTGCTGGGGGGTGCGGGTGCCGGGGCGGGAGGTGGTGCCGCCGGGGCGGGTGGTGTGTGCGGTGCCTGCG
GTGCTGGTGGCGCCGGGTGATCCAGGTGCGCTGGGTGTTCCGGGGGCGTGCGGTGCTCCGGGGGCTCCGGCGGGGGCCGGTGCGTCTGCCGTGCCTGC
GGTACCGGGCGTGCTCGATGCATCTGCCGCACGGGCAGCCCCTGCGGTACCCGCGGTACTCGCGCTGCCGGCCGAGCCCAGGGTGCCGGGCGTGTCGGC
GGTGCTGGTCGTATCCGCCGAGCCCGGTGAGTCCGCCGCGTGGGCGGCGTGGGCCGAACCCGCCGTCGCCGTATCGGCGGCGCCCGGCCCGCCTGCGGT
GCTCGCGGTACCGGCCGTATCCGGTGTGCCCGCCGCATGGACGGTGCCGGGCGTGCCGGGTGTGCTCGGTGCGTCCGCCGTACGGGCGGCGCCGGTTGT
ATCGGTCGCGCCCGCCGCGTGCGCGGCTCGTGCCGCGCACGGTGTGCCCGCCGCGCCCGGCGTACTGGCGGCCCCCAGCGAGGAGCCCGCGATCCGCAG
CGCCAGCGGCAGTCCGCCGCAGAGTTCGCGTACCCGGTCCTCGGCCTGGCCGTCGTACGGGTCGTCGAGCGAGCCGTTCGCGACGGAACGCAGCAACTCC
CCCGCTCCGGCGCCGTCCAGCGCCTCGACCGGGAGGTGGTGCACCCACGCCGGGGTGGTCTCCGGCAGGTCCAGCGGTCCGGGCGCGGTGACCAGGACC
AGGCTGTCGGAGCGTTCCGGGATCAGGGTGCGGACCTGCTCGGGGTCGGCCGCGTCGTCGAGGACCACGGTCACCGCGAGGCCGGTCAGATGCTGGTG
GTACACCTCGGCCAGCCGCCGTACCTGCTGTTCGGCGGAGGAACGTTCGCGGAAGAGCAACTGGTCCCGGGGCGCGCCGAGCCGGTTGAGGAGGTGGA
GCAGGGCGTCCCGGGTGGGGAGGGGACGGCCGCTGGCGCCGCGCAGATCGACCAGGCACGCCCCCGGAACAGGTCCTTGAGATGGTGGGACGCGCGC
AGGGCGAGTGCGGTGCGGCCGGAGCCCGCCGGGCCGTGCAGGACGACGACGGTGGGCCGGGTCTCGGTCGCGGCCCGCGCGGTCCGCACCCACTGGGC
GATCTGCTCCACCGCGTCCCTGCGGCCCGTGAACGGACCTTCCGGTTCCGGAAGCTGGGTGAAGGAGTGCGCGAGGGTGGAGCGGCGGCGGGCGAGCG
CGCCGCGGTCCCGGCCGCGCAACGGCGCCCCCGCGGGCTGCCGGTCGGCGCGGGTGGCGGCCGTGAGCATCCGCTGCTGGTCCAGGAAGGGCCGGATG
CCGCGCACCTCCAGCGCGGTGAGCCACTGGAGCCGCAACTGCTCGGTGCCGCCGGGCTGGCCGAGCGCTCCGGCCCTGCGGTGGGCGGCGGGCCAGTGG
GCGGCGGTCGCCTTGACGACGGTGGTCGCGGCTCCGGCCGCCGCGACGATCGCCCCGGCGCCCAGCGCGGTGCCGGTCGCCGTGCCGAGCGCGACATCG
GCGCCGACGGCGGCAGCCGCCGCGACCGCCGTCACCAGCGCCGGAGTTCCTATCTTCGCCCGGCTGAACTGCGCGGACAGCGGCTGCCTGGCGGCTTCGT
CCGCGCTCAGCGCCTCCTCGTACGCGCTGTATTCGTCGGCGGCGCCCGCCGCCATGCTGTCGAGCGCCGCCCGGCCGCGCGCGAGCAGCGTCTGCGCGTC
CGTCCGGCTGTCCGGGCGCCGGGTCTCCTCCTCCACGGCGCGTGCCAAGAGCCTTTCCGCTTCGGCCCGGTGACTGTCCCGCATCGGGGTCCCCCTCCCTC
GTCGGGTTCCGTTTACGTTGTCGAGTGTCCTGGGTGCGGCCCCCGGAAGCGAGGGAAGATCGCTGTGGCGTTCATGTCATACCGGTCGGGACGTCCGTCG
GCTCACCCCTCGGGGGGAAGGATCAGCGGCGGCCCGACGGGGAGCCAGTCCGGGGCCCAGCGCGGTGCGTTGTCCGAGGTGCAGCCGGGGGCGGCGC
CGTCCTGCGCGACGTTCGTGGGCGTGCCGATCCAGACGAAGTGCCAGAGCGCGAGCCCGGTGAGCACCGCCAGGAAGAACCCGGCGGGCACGACCGCC
CGCACCCACCGCGGCGCCCGCCGCACGGCCACCGCCGCGAGCGCCCACGAGAGCAGCCCGGCCGTGGCCCCGGCGATCACCGCCAGCGGCGTCTGCCAG
AACCACCAGTCGAACAGGCTGATGAACTCGCGCACCACGACGGAGCACTCCCGCCGCGCCGCCAGCGAGATCCGGTCGGTTCCGTAACCGGCGAGTCCGC
CGAGCAGGAGCGTGAGACAGCCGGGGAGCCAGGGTGGCCGCTGACGGGATTCCATGGGGGAAGAGACGACCCCGTACGCCGGTTCGGTTTCCGGGCCC
GGCCCCGTGCCCTCACGTCTCCCTGATGATGACGCGGCCCCCGCACAGCTTCGACCAGTCGTCGCGGTCGGAGGTCAGGACGACGACGGGCGCCGGAGC
GCGGAGTGCCAGTGCGGCGACGAGGGCGTCAATGGCGTACTTGTGTCCGTGCAGCCCACCGGCCTCCCGGAGCAGAGCGACCGCGGTCAGGGAGTCCTC
CTGGCTGACCGGTTCCACTCGCAGCCGGGAGAGAACCCAGTTCAGCCGTGAATTCTGGCCTTTGTGGTCCCC

Construct 2, 58% GC content (excluding handles):

GGCCGCACAGTAATTACGGTGCTGCGCTGGAGAAACAGGGTGTGGAAATCACGCTGATTTACAGCGGCAGCCATAAGGTGGATGGCAACCCCTACAGCC
ATCTTCCGGATGACGTCCGGGAGACACTGCAGTCCCGGATGGACGCAACCCGCCAGATGTTTGCGCAGAAGGTGTCGGCATATACCGGCCTGTCCGTGCA
GGTTGTGCTGGATACCGAGGCTGCAGTGTACAGCGGTCAGGAGGCCATTGATGCCGGACTGGCTGATGAACTTGTTAACAGCACCGATGCGATCACCGTC
ATGCGTGATGCACTGGATGCACGTAAATCCCGTCTCTCAGGAGGGCGAATGACCAAAGAGACTCAATCAACAACTGTTTCAGCCACTGCTTCGCAGGCTGA
CGTTACTGACGTGGTGCCAGCGACGGAGGGCGAGAACGCCAGCGCGGCGCAGCCGGACGTGAACGCGCAGATCACCGCAGCGGTTGCGGCAGAAAACA
GCCGCATTATGGGGATCCTCAACTGTGAGGAGGCTCACGGACGCGAAGAACAGGCACGCGTGCTGGCAGAAACCCCCGGTATGACCGTGAAAACGGCCC
GCCGCATTCTGGCCGCAGCACCACAGAGTGCACAGGCGCGCAGTGACACTGCGCTGGATCGTCTGATGCAGGGGGCACCGGCACCGCTGGCTGCAGGTA
ACCCGGCATCTGATGCCGTTAACGATTTGCTGAACACACCAGTGTAAGGGATGTTTATGACGAGCAAAGAAACCTTTACCCATTACCAGCCGCAGGGCAAC
AGTGACCCGGCTCATACCGCAACCGCGCCCGGCGGATTGAGTGCGAAAGCGCCTGCAATGACCCCGCTGATGCTGGACACCTCCAGCCGTAAGCTGGTTG
CGTGGGATGGCACCACCGACGGTGCTGCCGTTGGCATTCTTGCGGTTGCTGCTGACCAGACCAGCACCACGCTGACGTTCTACAAGTCCGGCACGTTCCGT
TATGAGGATGTGCTCTGGCCGGAGGCTGCCAGCGACGAGACGAAAAAACGGACCGCGTTTGCCGGAACGGCAATCAGCATCGTTTAACTTTACCCTTCAT
CACTAAAGGCCGCCTGTGCGGCTTTTTTTACGGGATTTTTTTATGTCGATGTACACAACCGCCCAACTGCTGGCGGCAAATGAGCAGAAATTTAAGTTTGAT
CCGCTGTTTCTGCGTCTCTTTTTCCGTGAGAGCTATCCCTTCACCACGGAGAAAGTCTATCTCTCACAAATTCCGGGACTGGTAAACATGGCGCTGTACGTTT
CGCCGATTGTTTCCGGTGAGGTTATCCGTTCCCGTGGCGGCTCCACCTCTGAATTTACGCCGGGATATGTCAAGCCGAAGCATGAAGTGAATCCGCAGATG
ACCCTGCGTCGCCTGCCGGATGAAGATCCGCAGAATCTGGCGGACCCGGCTTACCGCCGCCGTCGCATCATCATGCAGAACATGCGTGACGAAGAGCTGG
CCATTGCTCAGGTCGAAGAGATGCAGGCAGTTTCTGCCGTGCTTAAGGGCAAATACACCATGACCGGTGAAGCCTTCGATCCGGTTGAGGTGGATATGGG
CCGCAGTGAGGAGAATAACATCACGCAGTCCGGCGGCACGGAGTGGAGCAAGCGTGACAAGTCCACGTATGACCCGACCGACGATATCGAAGCCTACGC
GCTGAACGCCAGCGGTGTGGTGAATATCATCGTGTTCGATCCGAAAGGCTGGGCGCTGTTCCGTTCCTTCAAAGCCGTCAAGGAGAAGCTGGATACCCGT
CGTGGCTCTAATTCCGAGCTGGAGACAGCGGTGAAAGACCTGGGCAAAGCGGTGTCCTATAAGGGGATGTATGGCGATGTGGCCATCGTCGTGTATTCCG
GACAGTACGTGGAAAACGGCGTCAAAAAGAACTTCCTGCCGGACAACACGATGGTGCTGGGGAACACTCAGGCACGCGGTCTGCGCACCTATGGCTGCA

TTCAGGATGCGGACGCACAGCGCGAAGGCATTAACGCCTCTGCCCGTTACCCGAAAAACTGGGTGACCACCGGCGATCCGGCGCGTGAGTTCACCATGAT
TCAGTCAGCACCGCTGATGCTGCTGGCTGACCCTGATGAGTTCGTGTCCGTACAACTGGCGTAATCATGGCCCTTCGGGGCCATTGTTTCTCTGTGGAGGA
GTCCATGACGAAAGATGAACTGATTGCCCGTCTCCGCTCGCTGGGTGAACAACTGAACCGTGATGTCAGCCTGACGGGGACGAAAGAAGAACTGGCGCTC
CGTGTGGCAGAGCTGAAAGAGGAGCTTGATGACACGGATGAAACTGCCGGTCAGGACACCCCTCTCAGCCGGGAAAATGTGCTGACCGGACATGAAAAT
GAGGTGGGATCAGCGCAGCCGGATACCGTGATTCTGGATACGTCTGAACTGGTCACGGTCGTGGCACTGGTGAAGCTGCATACTGATGCACTTCACGCCA
CGCGGGATGAACCTGTGGCATTTGTGCTGCCGGGAACGGCGTTTCGTGTCTCTGCCGGTGTGGCAGCCGAAATGACAGAGCGCGGCCTGGCCAGAATGC
AATAACGGGAGGCGCTGTGGCTGATTTCGATAACCTGTTCGATGCTGCCATTGCCCGCGCCGATGAAACGATACGCGGGTACATGGGAACGTCAGCCACC
ATTACATCCGGTGAGCAGTCAGGTGCGGTGATACGTGGTGTTTTTGATGACCCTGAAAATATCAGCTATGCCGGACAGGGCGTGCGCGTTGAAGGCTCCA
GCCCGTCCCTGTTTGTCCGGACTGATGAGGTGCGGCAGCTGCGGCGTGGAGACACGCTGACCATCGGTGAGGAAAATTTCTGGGTAGATCGGGTTTCGCC
GGATGATGGCGGAAGTTGTCATCTCTGGCTTGGACGGGGCGTACCGCCTGCCGTTAACCGTCGCCGCTGAAAGGGGGATGTATGGCCATAAAAGGTCTTG
AGCAGGCCGTTGAAAACCTCAGCCGTATCAGCAAAACGGCGGTGCCTGGTGCCGCCGCAATGGCCATTAACCGCGTTGCTTCATCCGCGATATCGCAGTC
GGCGTCACAGGTTGCCCGTGAGACAAAGGTACGCCGGAAACTGGTAAAGGAAAGGGCCAGGCTGAAAAGGGCCACGGTCAAAAATCCGCAGGCCAGAA
TCAAAGTTAACCGGGGGGATTTGCCCGTAATCAAGCTGGGTAATGCGCGGGTTGTCCTTTCGCGCCGCAGGCGTCGTAAAAAGGGGCAGCGTTCATCCCT
GAAAGGTGGCGGCAGCGTGCTTGTGGTGGGTAACCGTCGTATTCCCGGCGCGTTTATTCAGCAACTGAAAAATGGCCGGTGGCATGTCATGCAGCGTGTG
GCTGGGAAAAACCGTTACCCCATTGATGTGGTGAAAATCCCGATGGCGGTGCCGCTGACCACGGCGTTTAAACAAAATATTGAGCGGATACGGCGTGAAC
GTCTTCCGAAAGAGCTGGGCTATGCGCTGCAGCATCAACTGAGGATGGTAATAAAGCGATGAAACATACTGAACTCCGTGCAGCCGTACTGGATGCACTG
GAGAAGCATGACACCGGGGCGACGTTTTTTGATGGTCGCCCCGCTGTTTTTGATGAGGCGGATTTTCCGGCAGTTGCCGTTTATCTCACCGGCGCTGAATA
CACGGGCGAAGAGCTGGACAGCGATACCTGGCAGGCGGAGCTGCATATCGAAGTTTTCCTGCCTGCTCAGGTGCCGGATTCAGAGCTGGATGCGTGGAT
GGAGTCCCGGATTTATCCGGTGATGAGCGATATCCCGGCACTGTCAGATTTGATCACCAGTATGGTGGCCAGCGGCTATGACTACCGGCGCGACGATGAT
GCGGGCTTGTGGAGTTCAGCCGATCTGACTTATGTCATTACCTATGAAATGTGAGGACGCTATGCCTGTACCAAATCCTACAATGCCGGTGAAAGGTGCCG
GGACCACCCTGTGGGTTTATAAGGGGAGCGGTGACCCTTACGCGAATCCGCTTTCAGACGTTGACTGGTCGCGTCTGGCAAAAGTTAAAGACCTGACGCC
CGGCGAACTGACCGCTGAGTCCTATGACGACAGCTATCTCGATGATGAAGATGCAGACTGGACTGCGACCGGGCAGGGGCAGAAATCTGCCGGAGATAC
CAGCTTCACGCTGGCGTGGATGCCCGGAGAGCAGGGGCAGCAGGCGCTGCTGGCGTGGTTTAATGAAGGCGATACCCGTGCCTATAAAATCCGCTTCCCG
AACGGCACGGTCGATGTGTTCCGTGGCTGGGTCAGCAGTATCGGTAAGGCGGTGACGGCGAAGGAAGTGATCACCCGCACGGTGAAAGTCACCAATGTG
GGACGTCCGTCGATGGCAGAAGATCGCAGCACGGTAACAGCGGCAACCGGCATGACCGTGACGCCTGCCAGCACCTCGGTGGTGAAAGGGCAGAGCACC
ACGCTGACCGTGGCCTTCCAGCCGGAGGGCGTAACCGACAAGAGCTTTCGTGCGGTGTCTGCGGATAAAACAAAAGCCACCGTGTCGGTCAGTGGTATGA
CCATCACCGTGAACGGCGTTGCTGCAGGCAAGGTCAACATTCCGGTTGTATCCGGTAATGGTGAGTTTGCTGCGGTTGCAGAAATTACCGTCACCGCCAGT
TAATCCGGAGAGTCAGCGATGTTCCTGAAAACCGAATCATTTGAACATAACGGTGTGACCGTCACGCTTTCTGAACTGTCAGCCCTGCAGCGCATTGAGCA
TCTCGCCCTGATGAAACGGCAGGCAGAACAGGCGGAGTCAGACAGCAACCGGAAGTTTACTGTGGAAGACGCCATCAGAACCGGCGCGTTTCTGGTGGC
GATGTCCCTGTGGCATAACCATCCGCAGAAGACGCAGATGCCGTCCATGAATGAAGCCGTTAAACAGATTGAGCAGGAAGTGCTTACCACCTGGCCCACG
GAGGCAATTTCTCATGCTGAAAACGTGGTGTACCGGCTGTCTGGTATGTATGAGTTTGTGGTGAATAATGCCCCTGAACAGACAGAGGACGCCGGGCCCG
CAGAGCCTGTTTCTGCGGGAAAGTGTTCGACGGTGAGCTGAGTTTTGCCCTGAAACTGGCGCGTGAGATGGGGCGACCCGACTGGCGTGCCATGCTTGCC
GGGATGTCATCCACGGAGTATGCCGACTGGCACCGCTTTTACAGTACCCATTATTTTCATGATGTTCTGCTGGATATGCACTTTTCCGGGCTGACGTACACC
GTGCTCAGCCTGTTTTTCAGCGATCCGGATATGCATCCGCTGGATTTCAGTCTGCTGAACCGGCGCGAGGCTGACGAAGAGCCTGAAGATGATGTGCTGAT
GCAGAAAGCGGCAGGGCTTGCCGGAGGTGTCCGCTTTGGCCCGGACGGGAATGAAGTTATCCCCGCTTCCCCGGATGTGGCGGACATGACGGAGGATGA
CGTAATGCTGATGACAGTATCAGAAGGGATCGCAGGAGGAGTCCGGTATGGCTGAACCGGTAGGCGATCTGGTCGTTGATTTGAGTCTGGATGCGGCCA
GATTTGACGAGCAGATGGCCAGAGTCAGGCGTCATTTTTCTGGTACGGAAAGTGATGCGAAAAAAACAGCGGCAGTCGTTGAACAGTCGCTGAGCCGAC
AGGCGCTGGCTGCACAGAAAGCGGGGATTTCCGTCGGGCAGTATAAAGCCGCCATGCGTATGCTGCCTGCACAGTTCACCGACGTGGCCACGCAGCTTGC
AGGCGGGCAAAGTCCGTGGCTGATCCTGCTGCAACAGGGGGGGCAGGTGAAGGACTCCTTCGGCGGGATGATCCCCATGTTCAGGGGGCTTGCCGGTGC
GATCACCCTGCCGATGGTGGGGGCCACCTCGCTGGCGGTGGCGACCGGTGCGCTGGCGTATGCCTGGTATCAGGGCAACTCAACCCTGTCCGATTTCAAC
AAAACGCTGGTCCTTTCCGGCAATCAGGCGGGACTGACGGCAGATCGTATGCTGGTCCTGTCCAGAGCCGGGCAGGCGGCAGGGCTGACGTTTAACCAG
ACCAGCGAGTCACTCAGCGCACTGGTTAAGGCGGGGGTAAGCGGTGAGGCTCAGATTGCGTCCATCAGCCAGAGTGTGGCGCGTTTCTCCTCTGCATCCG
GCGTGGAGGTGGACAAGGTCGCTGAAGCCTTCGGGAAGCTGACCACAGACCCGACGTCGGGGCTGACGGCGATGGCTCGCCAGTTCCATAACGTGTCGG
CGGAGCAGATTGCGTATGTTGCTCAGTTGCAGCGTTCCGGCGATGAAGCCGGGGCATTGCAGGCGGCGAACGAGGCCGCAACGAAAGGGTTTGATGACC
AGACCCGCCGCCTGAAAGAGAACATGGGCACGCTGGAGACCTGGGCAGACAGGACTGCGCGGGCATTCAAATCCATGTGGGATGCGGTGCTGGATATTG
GTCGTCCTGATACCGCGCAGGAGATGCTGATTAAGGCAGAGGCTGCGTATAAGAAAGCAGACGACATCTGGAATCTGCGCAAGGATGATTATTTTGTTAA
CGATGAAGCGCGGGCGCGTTACTGGGATGATCGTGAAAAGGCCCGTCTTGCGCTTGAAGCCGCCCGAAAGAAGGCTGAGCAGCAGACTCAACAGGACAA
AAATGCGCAGCAGCAGAGCGATACCGAAGCGTCACGGCTGAAATATACCGAAGAGGCGCAGAAGGCTTACGAACGGCTGCAGACGCCGCTGGAGAAAT
ATACCGCCCGTCAGGAAGAACTGAACAAGGCACTGAAAGACGGGAAAATCCTGCAGGCGGATTACAACACGCTGATGGCGGCGGCGAAAAAGGATTATG
AAGCGACGCTGAAAAAGCCGAAACAGTCCAGCGTGAAGGTGTCTGCGGGCGATCGTCAGGAAGACAGTGCTCATGCTGCCCTGCTGACGCTTCAGGCAG
AACTCCGGACGCTGGAGAAGCATGCCGGAGCAAATGAGAAAATCAGCCAGCAGCGCCGGGATTTGTGGAAGGCGGAGAGTCAGTTCGCGGTACTGGAG
GAGGCGGCGCAACGTCGCCAGCTGTCTGCACAGGAGAAATCCCTGCTGGCGCATAAAGATGAGACGCTGGAGTACAAACGCCAGCTGGCTGCACTTGGC
GACAAGGTTACGTATCAGGAGCGCCTGAACGCGCTGGCGCAGCAGGCGGATAAATTCGCACAGCAGCAACGGGCAAAACGGGCCGCCATTGATGCGAAA
AGCCGGGGGCTGACTGACCGGCAGGCAGAACGGGAAGCCACGGAACAGCGCCTGAAGGAACAGTATGGCGATAATCCGCTGGCGCTGAATAACGTCAT
GTCAGAGCAGAAAAAGACCTGGGCGGCTGAAGACCAGCTTCGCGGGAACTGGATGGCAGGCCTGAAGTCCGGCTGGAGTGAGTGGGAAGAGAGCGCCA
CGGACAGTATGTCGCAGGTAAAAAGTGCAGCCACGCAGACCTTTGATGGTATTGCACAGAATATGGCGGCGATGCTGACCGGCAGTGAGCAGAACTGGC
GCAGCTTCACCCGTTCCGTGCTGTCCATGATGACAGAAATTCTGCTTAAGCAGGCAATGGTGGGGATTGTCGGGAGTATCGGCAGCGCCATTGGCGGGGC
TGTTGGTGGCGGCGCATCCGCGTCAGGCGGTACAGCCATTCAGGCCGCTGCGGCGAAATTCCATTTTGCAACCGGAGGATTTACGGGAACCGGCGGCAAA
TATGAGCCAGCGGGGATTGTTCACCGTGGTGAGTTTGTCTTCACGAAGGAGGCAACCAGCCGGATTGGCGTGGGGAATCTTTACCGGCTGATGCGCGGCT
ATGCCACCGGCGGTTATGTCGGTACACCGGGCAGCATGGCAGACAGCCGGTCGCAGGCGTCCGGGACGTTTGAGCAGAATAACCATGTGGTGATTAACA
ACGACGGCACGAACGGGCAGATAGGTCCGGCTGCTCTGAAGGCGGTGTATGACATGGCCCGCAAGGGTGCCCGTGATGAAATTCAGACACAGATGCGTG
ATGGTGGCCTGTTCTCCGGAGGTGGACGATGAAGACCTTCCGCTGGAAAGTGAAACCCGGTATGGATGTGGCTTCGGTCCCTTCTGTAAGAAAGGTGCGC

TTTGGTGATGGCTATTCTCAGCGAGCGCCTGCCGGGCTGAATGCCAACCTGAAAACGTACAGCGTGACGCTTTCTGTCCCCCGTGAGGAGGCCACGGTACT
GGAGTCGTTTCTGGAAGAGCACGGGGGCTGGAAATCCTTTCTGTGGACGCCGCCTTATGAGTGGCGGCAGATAAAGGTGACCTGCGCAAAATGGTCGTC
GCGGGTCAGTATGCTGCGTGTTGAGTTCAGCGCAGAGTTTGAACAGGTGGTGAACTGATGCAGGATATCCGGCAGGAAACACTGAATGAATGCACCCGT
GCGGAGCAGTCGGCCAGCGTGGTGCTCTGGGAAATCGACCTGACAGAGGTCGGTGGAGAACGTTATTTTTTCTGTAATGAGCAGAACGAAAAAGGTGAG
CCGGTCACCTGGCAGGGGCGACAGTATCAGCCGTATCCCATTCAGGGGAGCGGTTTTGAACTGAATGGCAAAGGCACCAGTACGCGCCCCACGCTGACG
GTTTCTAACCTGTACGGTATGGTCACCGGGATGGCGGAAGATATGCAGAGTCTGGTCGGCGGAACGGTGGTCCGGCGTAAGGTTTACGCCCGTTTTCTGG
ATGCGGTGAACTTCGTCAACGGAAACAGTTACGCCGATCCGGAGCAGGAGGTGATCAGCCGCTGGCGCATTGAGCAGTGCAGCGAACTGAGCGCGGTGA
GTGCCTCCTTTGTACTGTCCACGCCGACGGAAACGGATGGCGCTGTTTTTCCGGGACGTATCATGCTGGCCAACACCTGCACCTGGACCTATCGCGGTGAC
GAGTGCGGTTATAGCGGTCCGGCTGTCGCGGATGAATATGACCAGCCAACGTCCGATATCACGAAGGATAAATGCAGCAAATGCCTGAGCGGTTGTAAGT
TCCGCAATAACGTCGGCAACTTTGGCGGCTTCCTTTCCATTAACAAACTTTCGCAGTAAATCCCATGACACAGACAGAATCAGCGATTCTGGCGCACGCCCG
GCGATGTGCGCCAGCGGAGTCGTGCGGCTTCGTGGTAAGCACGCCGGAGGGGGAAAGATATTTCCCCTGCGTGAATATCTCCGGTGAGCCGGAGGCTAT
TTCCGTATGTCGCCGGAAGACTGGCTGCAGGCAGAAATGCAGGGTGAGATTGTGGCGCTGGTCCACAGCCACCCCGGTGGTCTGCCCTGGCTGAGTGAG
GCCGACCGGCGGCTGCAGGTGCAGAGTGATTTGCCGTGGTGGCTGGTCTGCCGGGGGACGATTCATAAGTTCCGCTGTGTGCCGCATCTCACCGGGCGG
CGCTTTGAGCACGGTGTGACGGACTGTTACACACTGTTCCGGGATGCTTATCATCTGGCGGGGATTGAGATGCCGGACTTTCATCGTGAGGATGACTGGT
GGCGTAACGGCCAGAATCTCTATCTGGATAATCTGGAGGCGACGGGGCTGTATCAGGTGCCGTTGTCAGCGGCACAGCCGGGCGATGTGCTGCTGTGCTG
TTTTGGTTCATCAGTGCCGAATCACGCCGCAATTTACTGCGGCGACGGCGAGCTGCTGCACCATATTCCTGAACAACTGAGCAAACGAGAGAGGTACACCG
ACAAATGGCAGCGACGCACACACTCCCTCTGGCGTCACCGGGCATGGCGCGCATCTGCCTTTACGGGGATTTACAACGATTTGGTCGCCGCATCGACCTTC
GTGTGAAAACGGGGGCTGAAGCCATCCGGGCACTGGCCACACAGCTCCCGGCGTTTCGTCAGAAACTGAGCGACGGCTGGTATCAGGTACGGATTGCCG
GGCGGGACGTCAGCACGTCCGGGTTAACGGCGC

Construct 3, 42% asymmetric spread of GC content (excluding handles):

GGCCGCTCCGTGGTGGCACAGAGTACGGCAGACGCGAAGAAATCAGCCGGCGATGCCAGTGCATCAGCTGCTCAGGTCGCGGCCCTTGTGACTGATGCA
ACTGACTCAGCACGCGCCGCCAGCACGTCCGCCGGACAGGCTGCATCGTCAGCTCAGGAAGCGTCCTCCGGCGCAGAAGCGGCATCAGCAAAGGCCACT
GAAGCGGAAAAAAGTGCCGCAGCCGCAGAGTCCTCAAAAAACGCGGCGGCCACCAGTGCCGGTGCGGCGAAAACGTCAGAAACGAATGCTGCAGCGTC
ACAACAATCAGCCGCCACGTCTGCCTCCACCGCGGCCACGAAAGCGTCAGAGGCCGCCACTTCAGCACGAGATGCGGTGGCCTCAAAAGAGGCAGCAAA
ATCATCAGAAACGAACGCATCATCAAGTGCCGGTCGTGCAGCTTCCTCGGCAACGGCGGCAGAAAATTCTGCCAGGGCGGCAAAAACGTCCGAGACGAAT
GCCAGGTCATCTGAAACAGCAGCGGAACGGAGCGCCTCTGCCGCGGCAGACGCAAAAACAGCGGCGGCGGGGAGTGCGTCAACGGCATCCACGAAGGC
GACAGAGGCTGCGGGAAGTGCGGTATCAGCATCGCAGAGCAAAAGTGCGGCAGAAGCGGCGGCAATACGTGCAAAAAATTCGGCAAAACGTGCAGAAG
ATATAGCTTCAGCTGTCGCGCTTGAGGATGCGGACACAACGAGAAAGGGGATAGTGCAGCTCAGCAGTGCAACCAACAGCACGTCTGAAACGCTTGCTGC
AACGCCAAAGGCGGTTAAGGTGGTAATGGATGAAACGAACAGAAAAGCCCACTGGACAGTCCGGCACTGACCGGAACGCCAACAGCACCAACCGCGCTC
AGGGGAACAAACAATACCCAGATTGCGAACACCGCTTTTGTACTGGCCGCGATTGCAGATGTTATCGACGCGTCACCTGACGCACTGAATACGCTGAATGA
ACTGGCCGCAGCGCTCGGGAATGATCCAGATTTTGCTACCACCATGACTAACGCGCTTGCGGGTAAACAACCGAAGAATGCGACACTGACGGCGCTGGCA
GGGCTTTCCACGGCGAAAAATAAATTACCGTATTTTGCGGAAAATGATGCCGCCAGCCTGACTGAACTGACTCAGGTTGGCAGGGATATTCTGGCAAAAA
ATTCCGTTGCAGATGTTCTTGAATACCTTGGGGCCGGTGAGAATTCGGCCTTTCCGGCAGGTGCGCCGATCCCGTGGCCATCAGATATCGTTCCGTCTGGCT
ACGTCCTGATGCAGGGGCAGGCGTTTGACAAATCAGCCTACCCAAAACTTGCTGTCGCGTATCCATCGGGTGTGCTTCCTGATATGCGAGGCTGGACAATC
AAGGGGAAACCCGCCAGCGGTCGTGCTGTATTGTCTCAGGAACAGGATGGAATTAAGTCGCACACCCACAGTGCCAGTGCATCCGGTACGGATTTGGGG
ACGAAAACCACATCGTCGTTTGATTACGGGACGAAAACAACAGGCAGTTTCGATTACGGCACCAAATCGACGAATAACACGGGGGCTCATGCTCACAGTC
TGAGCGGTTCAACAGGGGCCGCGGGTGCTCATGCCCACACAAGTGGTTTAAGGATGAACAGTTCTGGCTGGAGTCAGTATGGAACAGCAACCATTACAG
GAAGTTTATCCACAGTTAAAGGAACCAGCACACAGGGTATTGCTTATTTATCGAAAACGGACAGTCAGGGCAGCCACAGTCACTCATTGTCCGGTACAGCC
GTGAGTGCCGGTGCACATGCGCATACAGTTGGTATTGGTGCGCACCAGCATCCGGTTGTTATCGGTGCTCATGCCCATTCTTTCAGTATTGGTTCACACGGA
CACACCATCACCGTTAACGCTGCGGGTAACGCGGAAAACACCGTCAAAAACATTGCATTTAACTATATTGTGAGGCTTGCATAATGGCATTCAGAATGAGT
GAACAACCACGGACCATAAAAATTTATAATCTGCTGGCCGGAACTAATGAATTTATTGGTGAAGGTGACGCATATATTCCGCCTCATACCGGTCTGCCTGCA
AACAGTACCGATATTGCACCGCCAGATATTCCGGCTGGCTTTGTGGCTGTTTTCAACAGTGATGAGGCATCGTGGCATCTCGTTGAAGACCATCGGGGTAA
AACCGTCTATGACGTGGCTTCCGGCGACGCGTTATTTATTTCTGAACTCGGTCCGTTACCGGAAAATTTTACCTGGTTATCGCCGGGAGGGGAATATCAGA
AGTGGAACGGCACAGCCTGGGTGAAGGATACGGAAGCAGAAAAACTGTTCCGGATCCGGGAGGCGGAAGAAACAAAAAAAAGCCTGATGCAGGTAGCC
AGTGAGCATATTGCGCCGCTTCAGGATGCTGCAGATCTGGAAATTGCAACGAAGGAAGAAACCTCGTTGCTGGAAGCCTGGAAGAAGTATCGGGTGTTGC
TGAACCGTGTTGATACATCAACTGCACCTGATATTGAGTGGCCTGCTGTCCCTGTTATGGAGTAATCGTTTTGTGATATGCCGCAGAAACGTTGTATGAAAT
AACGTTCTGCGGTTAGTTAGTATATTGTAAAGCTGAGTATTGGTTTATTTGGCGATTATTATCTTCAGGAGAATAATGGAAGTTCTATGACTCAATTGTTCAT
AGTGTTTACATCACCGCCAATTGCTTTTAAGACTGAACGCATGAAATATGGTTTTTCGTCATGTTTTGAGTCTGCTGTTGATATTTCTAAAGTCGGTTTTTTTT
CTTCGTTTTCTCTAACTATTTTCCATGAAATACATTTTTGATTATTATTTGAATCAATTCCAATTACCTGAAGTCTTTCATCTATAATTGGCATTGTATGTATTG
GTTTATTGGAGTAGATGCTTGCTTTTCTGAGCCATAGCTCTGATATCCAAATGAAGCCATAGGCATTTGTTATTTTGGCTCTGTCAGCTGCATAACGCCAAAA
AATATATTTATCTGCTTGATCTTCAAATGTTGTATTGATTAAATCAATTGGATGGAATTGTTTATCATAAAAAATTAATGTTTGAATGTGATAACCGTCCTTTA
AAAAAGTCGTTTCTGCAAGCTTGGCTGTATAGTCAACTAACTCTTCTGTCGAAGTGATATTTTTAGGCTTATCTACCAGTTTTAGACGCTCTTTAATATCTTCA
GGAATTATTTTATTGTCATATTGTATCATGCTAAATGACAATTTGCTTATGGAGTAATCTTTTAATTTTAAATAAGTTATTCTCCTGGCTTCATCAAATAAAGA
GTCGAATGATGTTGGCGAAATCACATCGTCACCCATTGGATTGTTTATTTGTATGCCAAGAGAGTTACAGCAGTTATACATTCTGCCATAGATTATAGCTAA
GGCATGTAATAATTCGTAATCTTTTAGCGTATTAGCGACCCATCGTCTTTCTGATTTAATAATAGATGATTCAGTTAAATATGAAGGTAATTTCTTTTGTGCA
AGTCTGACTAACTTTTTTATACCAATGTTTAACATACTTTCATTTGTAATAAACTCAATGTCATTTTCTTCAATGTAAGATGAAATAAGAGTAGCCTTTGCCTC
GCTATACATTTCTAAATCGCCTTGTTTTTCTATCGTATTGCGAGAATTTTTAGCCCAAGCCATTAATGGATCATTTTTCCATTTTTCAATAACATTATTGTTATA
CCAAATGTCATATCCTATAATCTGGTTTTTGTTTTTTTGAATAATAAATGTTACTGTTCTTGCGGTTTGGAGGAATTGATTCAAATTCAAGCGAAATAATTCAG
GGTCAAAATATGTATCAATGCAGCATTTGAGCAAGTGCGATAAATCTTTAAGTCTTCTTTCCCATGGTTTTTTAGTCATAAAACTCTCCATTTTGATAGGTTG
CATGCTAGATGCTGATATATTTTAGAGGTGATAAAATTAACTGCTTAACTGTCAATGTAATACAAGTTGTTTGATCTTTGCAATGATTCTTATCAGAAACCAT
ATAGTAAATTAGTTACACAGGAAATTTTTAATATTATTATTATCATTCATTATGTATTAAAATTAGAGTTGTGGCTTGGCTCTGCTAACACGTTGCTCATAGG

AGATATGGTAGAGCCGCAGACACGTCGTATGCAGGAACGTGCTGCGGCTGGCTGGTGAACTTCCGATAGTGCGGGTGTTGAATGATTTCCAGTTGCTACC
GATTTTACATATTTTTTGCATGAGAGAATTTGTACCACCTCCCACCGACCATCTATGACTGTACGCCACTGTCCCTAGGACTGCTATGTGCCGGAGCGGACAT
TACAAACGTCCTTCTCGGTGCATGCCACTGTTGCCAATGACCTGCCTAGGAATTGGTTAGCAAGTTACTACCGGATTTTGTAAAAACAGCCCTCCTCATATA
AAAAGTATTCGTTCACTTCCGATAAGCGTCGTAATTTTCTATCTTTCATCATATTCTAGATCCCTCTGAAAAAATCTTCCGAGTTTGCTAGGCACTGATACATA
ACTCTTTTCCAATAATTGGGGAAGTCATTCAAATCTATAATAGGTTTCAGATTTGCTTCAATAAATTCTGACTGTAGCTGCTGAAACGTTGCGGTTGAACTAT
ATTTCCTTATAACTTTTACGAAAGAGTTTCTTTGAGTAATCACTTCACTCAAGTGCTTCCCTGCCTCCAAACGATACCTGTTAGCAATATTTAATAGCTTGAAA
TGATGAAGAGCTCTGTGTTTGTCTTCCTGCCTCCAGTTCGCCGGGCATTCAACATAAAAACTGATAGCACCCGGAGTTCCGGAAACGAAATTTGCATATACC
CATTGCTCACGAAAAAAAATGTCCTTGTCGATATAGGGATGAATCGCTTGGTGTACCTCATCTACTGCGAAAACTTGACCTTTCTCTCCCATATTGCAGTCGC
GGCACGATGGAACTAAATTAATAGGCATCACCGAAAATTCAGGATAATGTGCAATAGGAAGAAAATGATCTATATTTTTTGTCTGTCCTATATCACCACAAA
ATGGACATTTTTCACCTGATGAAACAAGCATGTCATCGTAATATGTTCTAGCGGGTTTGTTTTTATCTCGGAGATTATTTTCATAAAGCTTTTCTAATTTAACC
TTTGTCAGGTTACCAACTACTAAGGTTGTAGGCTCAAGAGGGTGTGTCCTGTCGTAGGTAAATAACTGACCTGTCGAGCTTAATATTCTATATTGTTGTTCTT
TCTGCAAAAAAGTGGGGAAGTGAGTAATGAAATTATTTCTAACATTTATCTGCATCATACCTTCCGAGCATTTATTAAGCATTTCGCTATAAGTTCTCGCTGG
AAGAGGTAGTTTTTTCATTGTACTTTACCTTCATCTCTGTTCATTATCATCGCTTTTAAAACGGTTCGACCTTCTAATCCTATCTGACCATTATAATTTTTTAGA
ATGGTTTCATAAGAAAGCTCTGAATCAACGGACTGCGATAATAAGTGGTGGTATCCAGAATTTGTCACTTCAAGTAAAAACACCTCACGAGTTAAAACACC
TAAGTTCTCACCGAATGTCTCAATATCCGGACGGATAATATTTATTGCTTCTCTTGACCGTAGGACTTTCCACATGCAGGATTTTGGAACCTCTTGCAGTACT
ACTGGGGAATGAGTTGCAATTATTGCTACACCATTGCGTGCATCGAGTAAGTCGCTTAATGTTCGTAAAAAAGCAGAGAGCAAAGGTGGATGCAGATGAA
CCTCTGGTTCATCGAATAAAACTAATGACTTTTCGCCAACGACATCTACTAATCTTGTGATAGTAAATAAAACAATTGCATGTCCAGAGCTCATTCGAAGCA
GATATTTCTGGATATTGTCATAAAACAATTTAGTGAATTTATCATCGTCCACTTGAATCTGTGGTTCATTACGTCTTAACTCTTCATATTTAGAAATGAGGCTG
ATGAGTTCCATATTTGAAAAGTTTTCATCACTACTTAGTTTTTTGATAGCTTCAAGCCAGAGTTGTCTTTTTCTATCTACTCTCATACAACCAATAAATGCTGA
AATGAATTCTAAGCGGAGATCGCCTAGTGATTTTAAACTATTGCTGGCAGCATTCTTGAGTCCAATATAAAAGTATTGTGTACCTTTTGCTGGGTCAGGTTG
TTCTTTAGGAGGAGTAAAAGGATCAAATGCACTAAACGAAACTGAAACAAGCGATCGAAAATATCCCTTTGGGATTCTTGACTCGATAAGTCTATTATTTTC
AGAGAAAAAATATTCATTGTTTTCTGGGTTGGTGATTGCACCAATCATTCCATTCAAAATTGTTGTTTTACCACACCCATTCCGCCCGATAAAAGCATGAATG
TTCGTGCTGGGCATAGAATTAACCGTCACCTCAAAAGGTATAGTTAAATCACTGAATCCGGGAGCACTTTTTCTATTAAATGAAAAGTGGAAATCTGACAAT
TCTGGCAAACCATTTAACACACGTGCGAACTGTCCATGAATTTCTGAAAGAGTTACCCCTCTAAGTAATGAGGTGTTAAGGACGCTTTCATTTTCAATGTCG
GCTAATCGATTTGGCCATACTACTAAATCCTGAATAGCTTTAAGAAGGTTATGTTTAAAACCATCGCTTAATTTGCTGAGATTAACATAGTAGTCAATGCTTT
CACCTAAGGAAAAAAACATTTCAGGGAGTTGACTGAATTTTTTATCTATTAATGAATAAGTGCTTACTTCTTCTTTTTGACCTACAAAACCAATTTTAACATTT
CCGATATCGCATTTTTCACCATGCTCATCAAAGACAGTAAGATAAAACATTGTAACAAAGGAATAGTCATTCCAACCATCTGCTCGTAGGAATGCCTTATTTT
TTTCTACTGCAGGAATATACCCGCCTCTTTCAATAACACTAAACTCCAACATATAGTAACCCTTAATTTTATTAAAATAACCGCAATTTATTTGGCGGCAACAC
AGGATCTCTCTTTTAAGTTACTCTCTATTACATACGTTTTCCATCTAAAAATTAGTAGTATTGAACTTAACGGGGCATCGTATTGTAGTTTTCCATATTTAGCT
TTCTGCTTCCTTTTGGATAACCCACTGTTATTCATGTTGCATGGTGCACTGTTTATACCAACGATATAGTCTATTAATGCATATATAGTATCGCCGAACGATTA
GCTCTTCAGGCTTCTGAAGAAGCGTTTCAAGTACTAATAAGCCGATAGATAGCCACGGACTTCGTAGCCATTTTTCATAAGTGTTAACTTCCGCTCCTCGCTC
ATAACAGACATTCACTACAGTTATGGCGGAAAGGTATGCATGCTGGGTGTGGGGAAGTCGTGAAAGAAAAGAAGTCAGCTGCGTCGTTTGACATCACTGC
TATCTTCTTACTGGTTATGCAGGTCGTAGTGGGTGGCACACAAAGCTTTGCACTGGATTGCGAGGCTTTGTGCTTCTCTGGAGTGCGACAGGTTTGATGAC
AAAAAATTAGCGCAAGAAGACAAAAATCACCTTGCGCTAATGCTCTGTTACAGGTCACTAATACCATCTAAGTAGTTGATTCATAGTGACTGCATATGTTGT
GTTTTACAGTATTATGTAGTCTGTTTTTTATGCAAAATCTAATTTAATATATTGATATTTATATCATTTTACGTTTCTCGTTCAGCTTTTTTATACTAAGTTGGCA
TTATAAAAAAGCATTGCTTATCAATTTGTTGCAACGAACAGGTCACTATCAGTCAAAATAAAATCATTATTTGATTTCAATTTTGTCCCACTCCCTGCCTCTGT
CATCACGATACTGTGATGCCATGGTGTCCGACTTATGCCCGAGAAGATGTTGAGCAAACTTATCGCTTATCTGCTTCTCATAGAGTCTTGCAGACAAACTGC
GCAACTCGTGAAAGGTAGGCGGATCCCCTTCGAAGGAAAGACCTGATGCTTTTCGTGCGCGCATAAAATACCTTGATACTGTGCCGGATGAAAGCGGTTC
GCGACGAGTAGATGCAATTATGGTTTCTCCGCCAAGAATCTCTTTGCATTTATCAAGTGTTTCCTTCATTGATATTCCGAGAGCATCAATATGCAATGCTGTT
GGGATGGCAATTTTTACGCCTGTTTTGCTTTGCTCGACATAAAGATATCCATCTACGATATCAGACCACTTCATTTCGCATAAATCACCAACTCGTTGCCCGG
TAACAACAGCCAGTTCCATTGCAAGTCTGAGCCAACATGGTGATGATTCTGCTGCTTGATAAATTTTCAGGTATTCGTCAGCCGTAAGTCTTGATCTCCTTAC
CTCTGATTTTGCTGCGCGAGTGGCAGCGACATGGTTTGTTGTTATATGGCCTTCAGCTATTGCCTCTCGGAATGCATCGCTCAGTGTTGATCTGATTAACTTG
GCTGACGCCGCCTTGCCCTCGTCTATGTATCCATTGAGCATTGCCGCAATTTCTTTTGTGGTGATGTCTTCAAGTGGAGCATCAGGCAGACCCCTCCTTATTG
CTTTAATTTTGCTCATGTAATTTATGAGTGTCTTCTGCTTGATTCCTCTGCTGGCCAGGATTTTTTCGTAGCGATCAAGCCATGAATGTAACGTAACGGAATT
ATCACTGTTGATTCTCGCTGTCAGAGGCTTGTGTTTGTGTCCTGAAAATAACTCAATGTTGGCCTGTATAGCTTCAGTGATTGCGATTCGCCTGTCTCTGCCT
AATCCAAACTCTTTACCCGTCCTTGGGTCCCTGTAGCAGTAATATCCATTGTTTCTTATATAAAGGTTAGGGGGTAAATCCCGGCGCTCATGACTTCGCCTTC
TTCCCATTTCTGATCCTCTTCAAAAGGCCACCTGTTACTGGTCGATTTAAGTCAACCTTTACCGCTGATTCGTGGAACAGATACTCTCTTCCATCCTTAACCGG
AGGTGGGAATATCCTGCATTCCCGAACCCATCGACGAACTGTTTCAAGGCTTCTTGGACGTCGCTGGCGTGCGTTCCACTCCTGAAGTGTCAAGTACATCGC
AAAGTCTCCGCAATTACACGCAAGAAAAAACCGCCATCAGGCGGCTTGGTGTTCTTTCAGTTCTTCAATTCGAATATTGGTTACGTCTGCATGTGCTATCTG
CGCCCATATCATCCAGTGGTCGTAGCAGTCGTTGATGTTCTCCGCTTCGATAACTCTGTTGAATGGCTCTCCATTCCATTCTCCTGTGACTCGGAAGTGCATT
TATCATCTCCATAAAACAAAACCCGCCGTAGCGAGTTCAGATAAAATAAATCCCCGCGAGTGCGAGGATTGTTATGTAATATTGGGTTTAATCATCTATATG
TTTTGTACAGAGAGGGCAAGTATCGTTTCCACCGTACTCGTGATAATAATTTTGCACGGTATCAGTCATTTCTCGCACATTGCAGAATGGGGATTTGTCTTCA
TTAGACTTATAAACCTTCATGGAATATTTGTATGCCGACTCTATATCTATACCTTCATCTACATAAACACCTTCGTGATGTCTGCATGGAGACAAGACACCGG
ATCTGCACAACATTGATAACGCCCAATCTTTTTGCTCAGACTCTAACTCATTGATACTCATTTATAAACTCCTTGCAATGTATGTCGTTTCAGCTAAACGGTAT
CAGCAATGTTTATGTAAAGAAACAGTAAGATAATACTCAACCCGATGTTTGAGTACGGTCATCATCTGACACTACAGACTCTGGCATCGCTGTGAAGACGA
CGCGAAATTCAGCATTTTCACAAGCGTTATCTTTTACAAAACCGATCTCACTCTCCTTTGATGCGAATGCCAGCGTCAGACATCATATGCAGATACTCACCTG
CATCCTGAACCCATTGACCTCCAACCCCGTAATAGCGATGCGTAATGATGTCGATAGTTACTAACGGGTCTTGTTCGATTAACTGCCGCAGAAACTCTC

Construct 4, 42% GC content (excluding handles):

GGCCGCCGAAATTTTCTTCATAATAAAAGTTAAAAGCCGCATCTCTCGCTACAGCAATTTTCACTTGAGGTACGGACGGCTTTGTAAAGATAGGATCATTAA
TTTCTATTTTAGGAGCTAAAGCCAGTTCATATACTTTTTCGATATCAATTGTATCATGAACAAGCTTTCCTAACTGTTCAAAAAACGGTGTTAACTCTCCTCGT
TCAATCGACGGAATGAGCCCGAGGTGACGTTCAGGAATCGTAAGATCATCGTTTCTTTTCAAGTAGCCTACTACAGGGATATGACATTCCTGCTCAATCGCT

GCCTTAACAAGTTTATAATGTCCTTCACTTCCAACCCGGTTAGCAATCACCCCGACGATGTTCGCTTCGGGTAAAAATTGCTGAAACCCTTTTACAATCGCCG
CTGCACTTCTAGCCATACTAGCACAGTTGACAACTAAAAGGACAGGGCTATTGGTGATTAAGCTGATTTCTGCTGTTGTTCCTTCATTCGTTAACGGATTTTT
CCCATCGAAAAATCCCATAACTCCTTCCATGATGGAAATATCAGCATCTTCACTAGTACGAGCGACAATTTCTCTAACCATATCGTGTTCAAGCATCCAGCTG
TCTATATTTCGAGACGTTCGTTCTGTAACAGCCGTATGATACGTTGGATCAATATAATCGGGTCCGCATTTAAACCCTTGAACTCTGTAACCGGCTTGTCTTA
ATGCTGCCATCAGCCCGATTGTAAGGGTTGTTTTCCCTACGCCACTTCCGGTACCGGCAATGACGAGTCTTCTGTTTGACATGTTGTTCTCTCCTTTAGTCAT
ATGGAATAAGTGCCACTGAAATGGTTACATTCCCTGACTTTTTCTTCACAAGTTCAAGCTTATCAGCACCGCTGTATAACATAGCAGCAGGTTCACTAACGC
CATATGCACCTGTATATTTAAAGACAGTATCTGAAGGCTGTTGGATACTTATGCTGTTTAGCTCTTGGGGCGAATAATAGACAAACTCCCAGCCGTACTTAG
ACGCCACTTCTAACAGCCCTTCTTCATCTTTCTTTAACTCTATTGTACATAATGCTTTTACACTTTTCATTGAAAACTGAAGTTCAGCAAGCGTTTTTTCAATAA
CGGTTTCAATTTCAGCTGTACTTGTCCCTCGATTGCATCCCATCCCAATCGCTAATACTTTTGGCCGGTAAAGAACACCGTTTTCTAAGATTGCTTCTTCCTCT
TTTTTTAAATTGCGATGCGTTACGACCAAAGCAGCATGTGGCGATGCTTCAAGAGCTGTCTGAATCGATGAATATGTTTTAATGTTAGCTGGAACAGGGTG
CTCATAATGCCACCAGTTCTTTTCACCTGATTCTTGAACCACTGCTATTTCTTCTTCATTCACCACAGAAGCGCTGACAGGCGTTAGTTTTTCTGCTGATTCCC
ACACCCATCCAAAGCGCTTACCAAATAAATCAACCGGAATTGTTTTTTGCACATCGGATGCTGTTGTAATAACAGGATGAGCTTCAAGAGCTGCCGCAACTT
CACGCGTAAGCTCATTTGCTCCTCCGATATGACCTGAAAGCACGCTGATGACATTTTCGCCTTTATCATCAATGACAACAACCGCAGGATCCGTTTTCTTATC
TTTTAAAATAGGTGCAATCATACGTACAACTGCTCCCAGTGAGATAATAATAATAAGTCCTTTATATGATTCAAAAAGAGAAGGCAAAAGCATCCGAACGC
TTCCTGAAAACAGCTGAATGTTCTGCTCTTGTTCGTCTCCTTTTTCAAACTTGCTCATGTAATACACATCAGACTGCTGAAAAGTGCGGCCTAGATTTCTTGCA
ATTTCTACGCCGTGCTTCGTGATGGCCACGACAGCATAATCCCCGCGCTGTGTAATGAGGGCTTTTCTTCCTTCTTGGAGTTGAATCATTCCGACTTCACTCC
TCTTCGAAAGCCATGGGTAAATGTTTTATCATAAAGTTTTGAACGATAATCTTTGTCATGAATGTGTGGATCAAGTGCCCAGCCTGCCAAAATCATCGCCTG
CTTGCGAATGCCGTTTGTTCGCATAGCGTCATCGAGGTCTTTTACCGTTGTGCGAACAATTTTTTCATCTGGCCACGTTGCTTTATAGACTACTACAATCGGC
GTATCCTCACTCCAACCGGCGTTTATAAATTCTTTCATTACTTTTTTTGTGAGCGTCGCGCTTAAAAATAACGCAATCGTACATTTATGTTTTGCTAAATCGGT
TAATTTTTCAAACTCAGGCACAGGCGTTCTTCCTTCAGCACGAGTTAAAATAACCGTTTGAGTCAAATCTGGAATGGTTAATTCTGCCTCAGCCGCTGCTGCA
GCAGCGAATACGGAGCTGACGCCCGGAACGATTTCAATATCAACGCCTTCTCTTTTTAAAAGTACCATTTGTTCCATAATGGCTCCGTACATAGCAGGATCT
CCGGTATGAACTCTTACAACCATTTTTCCTTCACGCATGCGATCAAGCATCGTACCGACCATTTCTTCTAAATGCATACCCGCTGTTTTTAACACTTCAGCTCC
TGGTTTAGACTTAGCAACTAGCTCTTGACTTACCAGTGAGTCAGCGTACAAGACAACATCTGCTTGCTGAAGTAATTTTAAGCCTTTTACGGTAATTAAATCT
GGATCTCCGGGTCCAGCTCCGATTATGTATAACTTCATTTTCTCACCACCATTAGCGTTAAGTATTGTAAATCGGCTCCTTCTAGTTCTGAAGCTTTCCAGATA
ATTTCTTCATCTGACGTTACTTTTGTCACCACTGACGCTTTTTCAAGCAGATCAAGCTCTTTTAAAAGACCAACCATAAAATCGATGACTTTGGCCACTTTAAT
AAAAATGACGCAGTCGTTTTCCATTAATACTTTTTTCATTGTTTCATAATCTTCACGAGCAGGAACAATTGCTACGTGTTCATCACCGTCTGCTAACGGAATA
CCTAAACGAGATGCAGCTCCATTAATGGATGAAATACCAGGAATTACTTGAATCGGAGCTTCAGGGTGACGTTCTTGCATCAGGCGCATCATGTGAATAAA
CGTACTGTAAAGCATAGGGTCACCCTCTGTGACAAATGCTACATCTTTTCCTTCCTGGAGCTTTTCCCATACTCGTTCTACCGTTTCGGTCCATTTTCTTTCCA
AAATAGCAGGATCTTTTGTCATTGGAAACACAAGGCCAAGCATATCTTTTTCATTTGCACTGAAATAGACATCAATAATTTTTTGGGCATAGCTTTTGCTTCC
TTTTTGCTTTTTAGGATACGCAATAACAGGTGATTCTTTTAATTTACGAAATGCTTTTACTGTAATAAGTTCTGGATCTCCTGGTCCAACTCCAAGTCCGTACA
ATGTTCCAATCATATTCATTCTCCTCTTTTGGCTGTAATAATATAAATGGGGTTTAAAGCGTCAAATCGCGTTAAATGTAAAATGGGCTTGCTTCTCGAAATT
TGTGCAAGGGTAACAGCAGTCTCAAAGCCTCGTTCTTTAAAAGCTTTCATCGCTTCTGCTAAATTTTCAATCGTTACGGCATTTAATACGATGCGCCCTCCTG
AATTTAATCGACTGCAGCAAACATCTAAAATTGTTTCCATGCCTCCTGCTGTACCTCCAATAAACACCGCATCAGGATCAGCAAACTCATTGAGTCCTTCCGG
TGCTTTTCCGTGCACAGTGTGGGCGTCTACTCGAAATTTCGATAGATTTTCCCTGCAGTTTTCTAAGTCTGCTTCATTTTTTTCCACGGCAAAAATTTGTCCTT
CACGCGCAATTTTAGCCGCTTCAATTGCGACTGAACCCGTACATGTTCCGATATCCCATACGACGCTGTCTCTCTTTAGCTGCAGGGCGCTAATACTAAGTGT
ACGAACTTCTTTTTTGGTAATAAGTCCTTTGTCCGGCTTGCGTTGAATAAATTCATCGTCCTCTATTCCAAGAGGCCAGACTGGGCTCTTTTCTACCTGCTTTA
ATATCACGACATTAAGCGGAGAAAAGAGTTGATTCGCCGCCTCTTCCAGCGAAAGCAGCTGACAGCGCTCCGTTTCTCCACCTAGGTTCTCTGCCACAAACA
TCTTGTATTCTGTCATGCCGAATGATAATAAGTAGTTTGCCAGAGCACTAGGAGAATTTTGCTCATCGGTTAAAATCGCCACTTTTTTATAGCCATCAATGCG
CTGAGCCAGTCCCTTCATGCTTCTGCCGTGCACGCTTGTAAAATAAGCGTCTTGCCATCGCTCTTTTAGACGTGAGAATGCAAGCTGAATAGAACTTAAATA
AGGATATATTTCAACGTCTAGCTTCGTAGAAAGATAGCTCCCAATTCCGTAAAATAACGGGTCGCCTGAGGCTAACACAACCGCATTTCCTTCTTCGTTTTTT
AAACGTTCAACAAGAGGAGAAAGACCCCCTTCAATCGCTACTTTTTCTCCTTCGAAATCTTGAAAAAAATCCAGGTGACGCTTTCCTCCAATTAGGACATCA
CTTTCATACACCCACTGTTCATACATAGGAAGCAAACTTAACTTCCCATCGTCACCAATTCCAATAATTTTAATTGCCATGTTGCACCGCCTGTCCTAAAAAAT
CTCCTTTTAATGTGTAAAGAGACGTATCGACATCGATTCCGTCCCCTACTTCCTTTAATGCTGATAAACAGCAGTACTTGCACAGTTTTTCAAAAAACTGATG
ATGACCGCTTTGCGTCATTAAATCTCCCACTTGAGAAGCTGTGTTTGCACCTTTTATTTCTTCTACCAGTTCAGCAGAAGCGCCTGACTCAGAAGCCGCTTTT
GCTAAAAAATTAAAATCAATGGGTGCACTTTTTGAATGAACCATCATAACGCCTTGCGCGACTTTTGAAAACTTTCCCATCATGCCTACAAGCGACACTTTTT
TCATTCCTTGCTTTTTACATTGTTTTAAAGTAAATCCAACAAAATCTCCCATTTCGATAAATGCTTCTTCCGGAAGTTCGGGGAACTGTTTCATTCCATACTTTT
CGCTTCGGCCCCCTGTCGTAATGACAACGTGCTCACAATTACTCGCTTTAGCAACGCTGATGGCCTGCACGATACTTGCTTTATAAGCAGCTGTTGAGAACG
GAACGACAATCCCCCGCGTCCCCAGTATCGAAATGCCTCCTAAAATACCTAGACGCGCATTTAATGTCTTTTTCGCCATTTCTTCTCCTTCAGGAACCGAGAT
GACAATCTTAACGCCTTTTTGTATATTGTATTCAGCTAGAAGCTGCTCAGCCGTTTCTTTCAGCATCTTTCTTGGAACAGGGTTAATGGCTGCCTCACCGACA
GGAACTGGGAGGCCGGGCTTTGTCACACGCCCTACTCCCACTCCTCCATCAATGATAATGTCTGCACCATTACACCACGAAACTTCAGAAATAATCAGCGCT
CCGTGCGTGGCATCGGGATCGTCTCCAGCATCTTTAATGATGCTAGCTACAGCGCTGCTTGTGCGATACTCGCATTCTTCAAGATGAAATGTAGCAAAGCG
GCCGACAGGTAAGTAAATCGTTGACTCATCTTGCACTTCTCCTGAAATTAGCGTAAGCAGAGCTGCTCTCGTAGCTGCCGTTGCACAAGCGCCCGTCGTATA
GCCTTCTCTGAGTTTCTTAGGTTCTTTTGGTACTTCCTTCATGCGTTACACCTGATCTGCAAGAATTGAAATTGCATTTAATGCAGCAACCGTTACCGTACTGC
CGCCTTTTCGCCCGATGTTTGTAATAAACGGCACGTCAAGTTTTGCAAGTTCTTCTTTAGATTCAGCTGCTGAAACAAATCCTACCGGCAGACCAATAACAA
GCCCAGGCTTCGCTTCTCCTTCTTTAATCAAACGAATTAATTCAAGTAAAGCTGTGGGTGCGTTCCCAATTGCAAAAATTCCTCCGTCTGCTTCTTTGATGGC
TTTTCGCATAGAAATAATTGCACGCGTTGTATTTAAGCGCTTTGCTTCTTCCATTACGTCCGGATCTGAAATATAAACTTTGATTTCACCGCCGTGTTTTTCAA
TTCGCTGCTTGTTTGTTCCTACTTGCACCATTTGTACATCTGCAACCACTTGCTTGCCGCTGCGAATGGCTTTAATTCCTGCTTGAATCGCATCCGGATGAAAC
AGCATGCTGCGTCCAAGCTCAAAGTCTGCAGACGCATGAATTACGCGCTGAACAATTGGGTACTGTTCGTCTGTAAACGGATGAGGCCCAAGCTCTTCTGT
AATCATTTCAAAACTTTTTCCTTCAATTTGCTGAGGCTGAACAGTTAACGGTTTAAATTCTGTACGAAAATCCATCTCAATCAACTCCTAGTTTGGTTTACTAA
AGCATGAACCACTTCTTCAAACGTGGAATACACGGTCCCATACTCAATTTTCGGTCGCCCAATCATAATAATATCTAAGCCTAACTCTTTAGCTGCTTCCACTT
TTTCATCCACAGATCCAACTTTTCCGCTTTCTTTTGTGACCATGACTGTCACTCCGTATTGCTTATACAGCGCTCGATCAAACTCTTTTGTAAACGGCCCTTGA
ATAGCAATAATATTTTTTTGCGGAAATCCTAATTGCTGGCACTTTTCCATATTATCCAGCCTTGGAAGCATACGAGCAACAAGCCGTACATCTGAAAGAGGA

AGCAGCTTTTCTGTAAAAACCTGCAGCGTCTTGCTGCCTGTTGTCAGCATAATAACTCCTTTTTTCTCAGCTGCTACTTCTGCTGCTTCTTCATACGTAGAAAC
CATCGTCATATTATCATAGGTAAAAGCCTGCGAAGCCCGCTCATAGCGTATGTAAGGAATCGCTGTTTCAGCAGCCGCACCGATAGCATTTTTAGATGCTTC
TTCCGCAAAAGGATGACTTGCATCCACAATAGCTTTTACACGATGTTCGTTTACAAAATCCGTCATATCTTCTTTTGTGAGACGTCCAATTTTCACTTTCACTT
CTGCACGCTGTAATTCGATAGCTGCGTTTTCGGTTACAACCGTTGCCGTAACCTCATGACCTGCTTTTTTCACTTGCACGGCTAATGCACGTGCATCGCTAGT
TCCGGCTAACAATAAAATCATTTTAGCTCGCCGACCTTATCTTCACGGTGTTGATGGTCGTGATGGTGATCGTGGTGATGGTGTCCGTGATCATGGTCATGG
TCATGATCGTGATGATGGTCATGATCGTGATGATGGTCATGATCGTGATGATGGTGGTGATCAATATGCTCCATAATGCCGAGGCGATATTGACACGTATC
GCAGTTCATTTTCACTTCACCTTCTAATCCTTCTTCAGCACGTTCTTTTAAAATTGTCTGCAGTTTTGGGTGAAAACCGAAATAGCCGGCTAGCTTAAACTCAA
TGTTTTCATGCTGCATTTTATACTGCTTCACCATTTCTTCTAATCGCTTAATTAATACTCCTGTAAATAAGAAATACGGGAGAATAACCACTTTTTTAGCGCCC
AATTTCAAACATCGCTCCACGCCTTCATCAATCAGTGGTGCGGTCACGCCCATAAAAGAAGTTTCGACGATTTTATAATTTGTTTTTTCCCAAAGAAGGCGC
GTAATTTTATATAAATCACTGTTTGCATCAGGATCACTTCCTCCTCGCCCTAAGACAATAACAGCCGTGTCTTCAGCTGGAGTTTCTAAATTCTCTCCTGATTC
TTGTAATCTCGTTTTTAAAATTTCAAGGGCTTCTTCATGAACGCCAATAGGACGTCCGTATACGAAGTTTACGTGTGGATATTTTTCCTTTGCTTCATCTATAG
CCGCTGGAATATGTATTTTAGAGTGTCCAGCAGGCAAAAGCATAATTGGAATTACAACTACATCTTGTGCTCCTTTTGCCACACATGTATCAATGCCTTGACT
TACATTTGGGCGTTCAAATTCTAGAAAACACGTTTCTACTAAAATAGATGCGTCCCAATCATGCTTCATAGTCGAAATAAATTCCCGAATTTGATCGTTCCCT
TCAGGATCTCTGCTTCCATGACCGACAAATAAAACTGATTTCATATAATGTCCTCCTATTTTTTAATCTCCACACGGAGCTGGTTCTACTTTTATTTTTGGTGTT
ATATCTTGATAGTCGAATCCATAAACGTTCTTTACACGCTTAAAGAATTTATGAAATCGCTCATTAGGATGACCTTTTTCTTTATATTCATGGATAATGTTCTC
CACTATTTCAACAATTTCATCGGGTGCTATGCCTTCCGCCACAATTTGACCTGAATGCGCATTGCGTCCAACCGTTTTAGCTCCTAAAAATAAATCAAACGCA
CCTTTTCTATAGACAATGCCGATATCTTCTTGAACAGCGCCGTAACAAGCCATACCGCACCCATTGATACCAAGCTTCAGCTCTTTTGGCATGTCCATTCCCC
CGAGGCGCTTTTGTAATTCTTCCGTATGTGGAATAGCATCGCTTTTTTCTCCATCGCAAAAATCACACGCTTTGATTGTGAACACGTCTCCGACCGGAGATAA
TAAAAAGGAAGCTGCTTGGAGCTTTTCAATGATTATATCCGGCTGGGCCGTTGGAATTTGCAGTTTAATTTGATGGTCAGGTGTATACTCCATCGTACCTTT
TTCACCAACAACTTCCGCTAGTGTTGTCATTTGAACAGGCATGAATTTCTTATTTGCAAGTCCTGGACTAACTGCTACTTCAAAGATAGACTCGATTGGCTGG
TGTACGACCGCAGAAGTGGATTCACCTAAAATTGCTTGAAGCGCTTCTTCTGCAAGAGCTCGTGAAGTTGCTTTTTCTTCTCTTGTGTTCTGAACTGCTTCAA
TAGCTGAAGCTGTTTGCTTTACAGTCTCTTCTTGATCGAGAGCCCATGGTTCTGCTTCTTTTCGAAGTCGCTGATGCGGTCTTAGCGGCTGCTCCGTTTGGTT
GAGGGTATATTTCCGCTGATATCCTCGCGGTGTAATCATTAAATCGTCGTAAAAGAATGTAGATGAATTTCCGACAACAACGGTAGTCAGCATGCCTATTTC
ATGGTTTAACATGTCTTTTAAGTTGGTCATTACGACTTCTTCACGGTCACGATAAGCGCTTTTAACAAGACCAACAGGCGTATCAGGGGAACGGTACTTTAA
TAGAATCCTTTGCGCTTCAACAATTTGTCTTGTTCGTCTTCCGCTTTTTGGGTTGTAAAAAGCCACTACGAAATCTGCTTGTGCAGCTGCTTCAATTCTTTTTTC
GATTAGCTCCCAAGGCGTAAGGTGGTCACTTAAACTAATCGTACAAGCATCGTGCATCACGGGCGCTCCAAGGAGAGATGCGCATGAATTAATAGCCGAA
ATGCCAGGAATTACTTCCATCTCTACGCCCGTTTCTTTTTTCCAGCCTTTTTCAATCAACACTTCATACACAAGGCCTGCCATTCCGTATACTCCTGCATCTCCG
CTTGAAATGACAGCTACGGTTTTGCCCGCTTCAGCTTGCTTTACAGCTTCTTGTGCACGGCTTACTTCTTCGGTCATTCCCGTACTAATAATTTGCTGATTCGT
TAACAGTCCTTGAATTAGTTCCACATAGGTTTTATAACCGATGATCATATCACTTTCTTGAATGGCTTCTCTTGCTCGCTGTGTAATATGTTCAAAGCTTCCTG
GCCCAAATCCAATAACTAACAGTTTACCTTTCATGCTAGCTCCTCCTTTATGCGTTACTTTTTACTTTGTCAATTCCTTCTATGGAGTCACTGTTTTCCGGAGAA
ACGAAACCATTTTGATCACATTCATAAATAAGCGATTCCTTTATACTGCAGCCTCCTGCTAAATGCTCTTTGACAATCCGGTGAGCCACGGTTTCAGTGACCT
GCTTGTACCAATCTCCTGAAGGATAGACGATAGCTACAGGAGCATCTTTGCATCGTCCGTTACAGCGCGTACGCGTTGTATGAACCGTTTCATCCAAATCCA
GTTCCGTCACTTC

Construct 5, 39% GC content (excluding handles):

GGCCGCGGAAATCAAGATGAACTAATTTATACGTACAAAGTAAAAATTGGGAACAGCTGGAGCGAAGCGGTTGATCCATATGTACGAGCAACAACGGTG
AATGGAGATCGCGGAGTTGTCGTAGATTTGGCTAAGACAAATCCGAGAAAGTGGAGTACAAATAAGCCGAAATTTAAAAATCCAGAAGATGCGATTATTT
ATGAACTGCACGTACGAGATTTATCGTCTCAAAAAGAGAGCGGTATCAAAAATAAAGGAAAATATTTAGGCGTAGCGGAGTGGAATACGAAAGGACCAA
ATGGCGTAAAAACAGGACTCAGTCATATTAAAGATTTAGGTGTGACGCACGTTCAGTTCCTGCCTATTTACGATTATCGTACGGTGGATGAAACGAAATTA
AACGAGCCGCAGTTTAACTGGGGATATGATCCAAAAAATTACAACGTCCCAGAAGGTTCCTATTCAACAAACCCATATAATCCTAAGACGAGAATTATTGA
GCTCAAACAAATGATTCAAACGCTTCATGATAAGCAGCTTCGTATGATAATGGACGTTGTTTATAATCATGTGTATGCAGTGAGTGAGCATAGCTTTGATAA
ACTAGTTCCAGGCTACTACTTCCGTTATAAAGAAGATGGAACTCTATCCAACGGAACAGGTGTTGGAAACGACACGGCGTCTGAACGGAAAATGGTTCGG
AAGTTTATTGTAGATTCTGTTGCGTATTGGGCAAAAGAGTACCACATTGACGGGTTTCGATTTGATTTAATGGGGATTCACGATACAAAGACAATGAATGA
AGTGAGAAAGAAGTTAGATGAAATTGATCCTTCTATTATCGTCTTAGGAGAAGGCTGGGATCTAGAAACCGAGCTTGATGCTAAGCTAAAAGCTAATCAG
AAAAACGCTCAGGATATGAAGCGCATCGCTCACTTTAATGACGGTATGCGAGATGGTCTTAAAGGCAGCGTATTTTTTGATCGTGACAACGGATTTGTCAA
TGGAAAACAGGGTCAAGAAAAGCTCATACAGCAAAGTATAGCAGCTGGAATTGATTATGATCATTCAACTGCCACGTATGAAGACCCAGATCAAGTTGTG
ACGTATGTAGAAGCACACGATAACCATACATTATGGGATAAACTTCAGCTGAAGAACCCTGCTGAAACCGAACAAACGAAAAAGCAAATGCATAAGCTTG
CTTCTTCTATTATTTTAACATCTCAAGGAATGAACTTTATACATGCAGGTCAAGAGTTTATGAGAACAAAAGGCGGAGATCATAATAGCTATCAGTCACCCG
ATTCTGTCAATCAGCTAGATTGGAAGCGCCGAGCAGCTTTCAGCCAAGAAGTAGATTATATGAAGGGACTTATTGCGCTTCGAAAGCAATATTCCTCATTTA
GAATGACGAGTGCACAGGCTATTCATCAAAATTTACGCTTTGTTAATGCATCAGCAAACGTAGTAGGCTATACGTTAAATGCTAAAGCCAATAAAGATAAA
GCAAACGAAATAATGGTGATTCATAATGCAAATAAACAAGCTCAAACGGTTCATCTACCTTCTAACAGAACGTGGAGATTACTTGTTGACGGCGAGCAGGC
AGGAACAAAAACGCTCCGTACGGTGAAGGGAAATACAGTAAACGTTTCGCCTCTTTCGACATTTGTGCTAGTAAGATAAATTTTAGACAACTAGAATATAT
GTAGAAAAATTCAGAATATTGTATGCAAAATTGTTGATTTTACTAGATAACATGGTAAAATATTCATTGTTAGCGTTTTCAAAATTCGTGGGGAGGTATGTA
TTTGATGCAAAATCTTGGAGCGAAAAAAGTTGAAATGAGTCCGAAAGTAGTGGAGTTTTTAAAAGGAGTTAAACAGCTATACATTAACGGCGAATGGGTA
GACTCTGTTTCAGGAAAAACGTTTGAAACAGTAAATCCAGCAACGGGTGAAAAGTTGGCAGATGTAGCTGAAGCAAATCCAGAGGATATTGATCGTGCCG
TCAGAGCGGCTAGAGAAGCCTTTGATCATGGACCTTGGACAAAGATGTCAGCAGCTGAAAGAAGTCGACTTATTTATAAATTAGCTGATTTAATGGAAACA
CATCAATTGGAGCTTGCGCAGCTTGAAACGCTTGATAACGGAAAACCAATTCGTGAAACATCAAATGCAGACATTCCATTAGCAATTGAACATTTCCGCTAC
TTTGCTGGGTGGTCAACTAAAATGGTTGGACAAACTATTCCTGTGCAAGGAGCGTATTTTAACTATACAAGATATGAACCAGTAGGGGTTGTTGGCCAAAT
TATTCCGTGGAACTTTCCACTTTTAATGGCCGCTTGGAAGCTCGGAGCCGCTTTGGCAACTGGATGTACAATTGTGCTCAAGCCAGCAGAGCAAACGCCGC
TTTCTGCACTTTACTTAGCTCGACTAGCCGATGAAGCAGGGTTCCCAAAAGGCGTGCTAAACATTGTGCCAGGCTACGGGAAAACAGCAGGTGAACCGCTC
GTTCATCATGATCTTGTCGATAAAATTGCTTTTACAGGATCTACTGCTGTCGGAAAAGCAATTATGAAGCAAGCTAGCGACTCCTTAAAGCGTGTGACGTTA
GAGCTTGGAGGTAAATCACCAAACATTATCCTCCCGGATGCAGACTTAACAAAAGCAGTGCCTGGTGCGCTTTCAGGTATTATGTTTAATCAAGGACAAGT

ATGTTGTGCTGGAAGTCGTCTGTATGTCCAAAAAAGCTTATATGATAATGTCGTAGCTGATTTAGTTTCTCAAGCGAAATCTATTAAACAAGGAAATGGATT
AGCAGATGAGACGACAATGGGTCCGCTTGTATCTGCGCAGCAGCAAAAACGAGTGAAAACCTATATTGATAAAGGAATTGAAGAAGGAGCGGAAGTACT
CGCAGGAGGAAACATTCCATTTGATCAAGGCTATTTTGTAGAACCGACTATCTTTGCAGATGTCGATCATTCGATGACGATTGCTCGTGAAGAAATCTTCGG
TCCGGTTGTAGCTGCTATGCCGTTTGACGATCTTGATGATTTGATTGCCAAAGCAAATGATACAGCATATGGTCTAGCAGCGGGCGTCTGGACACAGGATT
TAAAGAAAGCACACTATATTGCTCATGGTATTAAAGCGGGTACAGTTTGGGTGAACTGTTACAACGTATTTGATGCAGCAAGCCCGTTTGGAGGCTATAAA
CAGTCGGGAATTGGTCGTGAAATGGGAAGCTATGCGCTTGATAATTACACTGAAGTGAAAAGCGTATGGATCAATATGGAATAATAAGTAAGATTTTTTGT
GAAAATCAATAGCATATCTTTGTGTGAAAAAGAGTAGTCTTTAGTGGACTACTCTTTTATTTGTTTAAGTCCCGCATTTTTAGAAAAGGAGTGATAACATTGC
CGGTTATACAGGGCCCCTCGTCGTATAAGCTTACCGGTCACCTGCAAAAGTTTAGAGAGAACCCACTGGGTTTTTTGGAAAACTTGACTCAGTATGGAGAA
ATTGCAACATTTAGAGTTGCGCACAAACGCTTTTATGTAACGAGAGACCCTCAACTTATTAAAGATGTAGTCATTACAAACAGCAAGGCGTTTCAAAAAATT
AAACTAACGCACATGTTTAAAACGCTGCTAGGCGAAGAAATGTTATGGACAGATGAAGCATTATACATGAGCCCCATTCAACCCTCACAACTTAAACAACA
TCTAACTTATAATAAGGAAGTAATCGCAAAAATAATTGAAAAGCATACGGAAACGTGGGAAGAAGGGCAGCTTCGAACGATAGTAAAAGACATAAGACA
GATAGTCGTAGCTGTACTCCTTCAACTTGTATTTGGCATTTCGATTCAGGATAAAGATAAAATTCACTATGTTCAAGCGCTTATGAGAAAAAAAGAAAAGTT
AGGCAAAATTTATATTCGTCTACCTTTGCACCAGCCTGACTCAGATGAGCAGCTAGAACAACTTCTTTTTGAACGTGTTCAAATGCGTGTTCAAAACAAAAC
CGAAGGAAATGATTTACTGGAGTATATATTGAATTCTTGCGGAGAAGAAATCGATGAAAGAGAAATATACGAACAGCTAAAATCTATATTCCTTTCGATGT
ATGAAATGATTACGCACGTATGCAGCTGGTCTATTCATTTACTGTCTCAAAATACTAGGGAGCATCTTCAGCTGCATAAAGAAATTCAAGCCTATGCTAGCG
GCGAATCACTTTCAACCAAAAACCTGACCTATATGCGTAAAATAATTGCTGAAAGCATGAGGTTGTATCCGCCTCTTTGGCTATTTGGGCGTCAAGCACGTG
AAGATATACAAATAGATGGCTATAGTATTAAAAAAGGGGAGATTATGTTAATTAGCCCTTATATGATGCATCGCCATGAAGACTATTTTTTAGAGCCAAGT
GAATTTTTACCTGACCGTTTCGAAAAAGGAGGGTCCATTGATGTCCCAAGCTATATGTATATGCCGCTTGGGATTGAGCATCAAGCGGAAAGGGGCATGG
ACTATATCACTGAAATAGTAACCATTTTTATGTCAGAAATGACAAAGCGCTTTTTATTTCAGCTGACTAAGCCCGAATCTATTGCTCCGATGGCGGGCGTAA
TGCTGAATATGAAAGAAGAACTGAATGTGAATGTTCACAAAGTTCACACGCAGTCTTGAATGAATGGGAATACTTTCCCTCTTTATATCAGGCTGTCAATTT
ATTGACACATCCTGCATTGAGATTTATCATAGGGTAATATTTGATAGAGATTACTGAAGGATTTTTAGAGGGGACTATGGATAAAATAATTAAACAGTTGG
TGTCGCTAGTTGAAACATCATTACTAACGCAAGTTACGATTCAAAGTGAAAGTGACTATGATCCAGTTGAAGTTACGAACAAAACAAAAGAGTGGGTACTT
ATTGGTAAAGGAAACTATGCCGGTGTGTTTACGCACGAAAATTATCCTGAATTTGTCGTGAAAGTATACGGAAGAGAAATTTATGGTGTAAAAAAAGAAG
CGCAAGTGTATAGAAAGCTGGGCGTTCACCCAGCTTATTCTCAACTTTTTCATGAGGGAAAGACGTACTTGATTTTAAAAAGGCTTACAGGCGTTACACTGT
ATGATGCCGTCCAGCGGGGCATAAAGATTCCAGAACAAGTCATAAAAGATGTGAATAAAGCTTTGGACTATGCAAGAAGCCAGGGTTTAAACCCATATGA
TGTGCATGGTAAAAATGTCATGATGAAAGATGGACGAGGGTATGTGGTGGATATCTCTGACTTTTATAAAGAAGGTATTGATAAAAAATGGACAGACCTT
GTAAAAGCTTATTACACCTTTTATCCGTTTATTGATAAGTACCATATCCGCTTGCCGTACCCGCTTTTAAACGTTATCAGAAAAGCATATCGTCTATATCGTAG
AATCAAATCAAAAAGGAGTTTCCGCTAAAGGAAACTCCTTTTTAAGATGAAATACAGCGCAATACATGCTGCTGCTCAAAAGTTGGTTTTAATTTGAATAGA
ATGGTTTGTAGTATTTCAGATTGATAGACAAATTTTAGCGTATGTTTAAGGGAAAGAGGATGTAAGAAAGAACGTATTCTTTTATTAGCATAACGTATCACC
TGTTCTTCATAAAATAAAGTACGTGCGGAGATTTTAGCGTGAATGTCTTTTGTTAGTTCGATTGTAAACTGGATAATCATTAAATCAGTGTCTTTACAGTATT
TTTCTAGTAGCATGATATCACCTCCATTTTGTCAACTCATTTACATAATCTTAATATAAAATTTACAAATGTCAATAAAAGACGGAAAAATAAATGGTAAGAC
TGTTCTTTTTATATGTTGGATAAACAAGAACGTGATAGAGGTGAGTAAAGTTGAACATTAACGTCGGATTTGCAATTTTAGCTGATATTGATAATAAGATGA
CGGCTGCTATTTATGTGGAAAATCAAATTGTAGCGATTATAGCTGGACCTTCAGATATTCTTTATGAAAAGTTAAAAAAAGTGTTTTTATAATAGAGAAAAG
CTGTAATTGTAAATATGATTCATAAAAAGCTTAAAAATTCTATGAAATAGTCGAAATAAAAGATGAGGATAAAATACACAAAAAGAAAGAACAGGAAGTT
AAGGATAGGAGAATTACATATGGATAAAACATCTTTTATTGGACTGATTTTAGGAATAGCCTCTCTTATTGTAGGCATGTTTTTCAAAGGGGTTAATCCATC
TGTGTTAGGAAATCCGGCTGCTATTTTAATTATTATTGTCGGAACCGTCGGAGCAGTAGTGATTGCATTTCCTTCAAATGAAATCAAGAGAGTCCCAAAACT
GTTTGGTGTCCTGTTTAAAGAACAAAAAATGCTGCAGCCCGTTGATTTAGTATCCATGTTTTCTGAATGGGGACAAGTTGTTCGAAAAGAAGGGTTACTATC
ACTTGAAGCCCAAATTATTGATGTAGATGACCCTTTCTTAAAAAATGGTTTGAATTTAGCTATTGATGGTCAAAGTGCGGATTATATACGAGACGTGTTGTC
TGAAGAAATTGATGCTATGGAAGAAAGACATCAAACGGGTGCAAGTATTTTCGCTTTAGCAGGTACATATGCTCCAACTCTGGGTGTACTTGGAGCTGTTA
TAGGGTTAATTGCTGCGCTTGGGAACATGGAAGATACAGATACGCTAGGACATGCGATCAGCGCGGCGTTTGTTGCTACGCTTCTTGGTATTTTTACTGGG
TATGTACTATGGCATCCGTTTGCAAATAAACTAAAGCGTAAATCGAAACATGAAGTAAAGGTAAAGTATATGATGATCGAAGGAGTGCTTTCACTTTTAGA
AGGAGAAACACCAAAAGTAATTGAGCAAAAGCTGGCTTCTTATTTGCCAACAGCGGAACGTAAAAAGCTTCTTCAAGAAAGTGAAGTGGGCATAAATGAG
TAGACGCAGAAAACGAAAGCATGAAGAGGACCATGTTGACGAAAGCTGGCTGCTTCCTTACTCAGACTTGCTTACACTTTTACTTGCTTTATTCATTGTATT
GTTTGCAATGAGCTCAGTGGATGCGCAGAAATTTAAGAACCTTTCTCGCGCATTTAATGAAGCGTTCGTTGGAGGAACGGGCGTGATGGAATTTCAATCTC
TGCAGGAATCTGAAGAAGCGGTTCAGCCTTCAAATACTCCTGCAGCTAAAAGTACGGAAAAAACAAATGAAGTGGAGACAGCCCCCAATCAGTCTCCTGC
TTCTTCACAATCGTTGACGAGCGAACAAAAGGAGCAAACCATTCGAGAAGCTGATCAAGAAGAGCTTCAGCAAATTCAATCTAAAATTAATGCGTATATTA
AAAAGAAAAACTTAACAAATCAATTACAGACTTCGCTAACCGAGGAAGGTCTCTTAATTTCCATTCGAGACAATGTATTGTTTGATTCGGGAAGAGCACAA
GTTCGGAGTGAAGATACAAAAATCGCAAATGATATTTCAGAGCTTTTGGTGATTGATCCTCCTCGTAATATTATTATTAGCGGTCACACCGATAACGTACCT
ATTCGCAACGCTGGTTTTGAATCCAACTGGGAGTTAAGCGTGATGAGAGCAGTTAACTTTATGAAAGTTATCTTAAAAAACGATAAGCTTAATCCAAGCAT
GTTCAGTGCAAAAGGATTTGGAGAGTTCAAACCGGTGACATCTAACAATACAGCAGAGGGACAAGCGAAAAATAGGAGGGTTGAAATATTGATTACTCCT
CGTACAATTAAACAGCCGTAAAAGCAGTCCTGCTTTTACGGCTGTTTTTCTATGTGTTATAAAGCTAAGAGTCTTCTCTTTTCAAAGTTTCCTCGTACTAACTT
GAGATCCATTCCATCTAAAATCAAGTCAATGAGAGCGCCTTTAAAGACATCCGCTGTTTTTTTCTTATCATTGGACTGCAAATATTGCACGCGATACCACTTG
TCTTCAACTTGAAAGTACATAAACCGTCCGTGCGTAAAATCAATCCAAAACTTTGTAGGTTGTCCATTGAGATTTTTATAAACATATCCCCAGCTTCGTCCTT
GGTGAGCGTCCTTCATTTTAGGGCGAGCTGTATTAAATACTTCGTAATATACTTTGTGCTTAATATCATAGAGCATCATGTGACAGTCCTCCTATCTATAGAC
GTGCTAGCAGGTAAAGATATATTTGTATACAACAAGTATACTCAGCTGAATGGAATCGTAGACTATTTTTTTGAGACAAGCAGAAACGCAGTTAAATGAAA
AAGACAGTTTAAATTAGCTCAATCATTCCGCTTTTTTTATACTGTAAGTAAATGCGTTAAATAAGGAGGAAAAAATGAGACGAGTAGGAATAGTAGGTGCT
GGAATGACGGGCCTTACAGCAGCGGCTGAATTAAAAAAAGAAGGAATAGAAGTCTTTCTTCTTGATAAGGGTAAAAGTGTAGGGGGACGAATGGCTACT
CGAAGAGTAGGAGATGGAAAAGCAGATCACGGAGCTCAATTTTTTACAGTGAGATCCGATGAATTTCAACAAGACGTAAATAAATGGATAGCTGATAGAA
AGGTTAAGAAGTGGTTTGGTGACCATCACCCTCGGTATCAAAGTATGAATGGCATGAATGCGCTAGCTAAATATTTAGCTGAAGATCTACGCGTATACGTG
AATCGAAAAGTTCAAGCCATTGATTTTCAAAACGGAAGGTATCAGCTGTATACAGAAGAAAATGAGATTTTTGAAGCGACTGATATCATTCTTACACCTCCG
TCTCCTCAGGTTATTGAGGTACTTAATAATAGTAAGCTGCAAACGGACCAATCAATTCTTAATACACTGAAATTTAGTCCCTGCTTGGTTGCTATCGTGGAGC
TTTACACAGAGATGCTGTATGGTGATCATGGTCAAATCACGAATCCTTCTAGCACAATCCAAAGAATCGTTAATCATGAGCAAAAAGGAATATCAAAGACG

CCCATTCTTAGCATATATATGAATAAAGACTGGTCAGAAAAACATTTTGATGAACATGAACATGAGCTGCTGCGGGCGATAAAAAATGAAATAAAAGAATG
GATAGGTGCAAATCATATTAAAAGCATTCAGTTAAAGAAATGGCGCTATGCAGAGGTTGAACAAGTTCTTCATCAGCCCTTTGCTAAAATTATGCCGTCGCT
ACTGGTGGCAGGAGATGCTTTTTTAAGAAGGGAAGACGAAACAAATCACTCACGGTTAGAGAGCGCATATTTGTCAGGGAAATCGGCAGCCGCTGAATTA
ATGGGAAAGAACATCTAGAAAAAGGAGGGACCAAGAAGTGAAAAATAATTGGATAATGAAACAAACGTGGAAAGATTTACTGTTTATTCATTGGCCTGTA
GATCCGACATTTTTGGCTTCTTTATTGCCTAGTCAGTTAGAGCCTGACACCTATGATGGTCAAGGATGGATTGCACTTGTGCCTTTTACGATGACAGACATTC
GATTCAAAGGAACGCCCGCAGTTCCCATCTTTTCACGGCTATATGAGCTGAATGTGCGTACATATGTTACATATAAAGGGGAAAAAGGCGTTTATTTTTTTA
GTTTAGACGCAAGCAATCCGCTAGGGGTATGGATTGCTCGTCAGTTTTTTCATTTGCCTTATTACCACGCTACTATGACATTCAACAAAACTCAGAACCATAT
TTCTTTTCAGTCAGTTCGAACGCACAGAGACTCAAAAAAAGAGCGAGTAAAGCTTACATATAGAGGTATTTCTCCGTCCTATCGCAGTAGAAAAGGAGAAT
TAGCTCATTGGCTTACAGAGCGCTACTGCTTATTTACCACTCATCAAGGAAATGTCTACAGAGGAGACCTTTATCATGATCCTTGGGAGCTGCAAAAAGGG
GAGTGTGAAATAAGAGATGATTCTATTACTCCGCCTTTTTTGCGTCCCGCGGACAATCGCGATTTAATTGTGCACTATGCAAAC

Construct 6, 38% GC content (excluding handles):

GGCCGCCGCTAAACACTGTACAATTCGCAGCCGGTTAGGATCACTCAACGCATGTAAAATAGGCGTTAATTTAATTTCAGATTTCGGTGGTTGATAAGGTTT
TCTCATGCTTAAAATATAACACAGCTTCTTTTGTGTATCAATGTTCGATAACTTTCGAACTAAATTCCTTGCACTGTTATTTAATCGGTGTTATAGTTTCATTGT
TCGATGATATTCGAACTTATCTTCATTTTAATCGTAAATTGAATGAAAGATTAAAAACAAAACAAATAATATAAAGCACCATCATTATTAAAGGAGGAATCA
CCCATGATTCATTTACAAGCTTCAATGAAAGTTAACCCAGCAAAAAGAGAAGAATTTTTAGAAAACGTACAAGAACTAATTAAGCATTCTTTACAAGAAGAA
GGAAACAGCAACTATCAACTATTTGAAGATGCTTTTACACCTAACACATTTATGATGATTGAAGAGTGGAAAAGCGAAGAAGCAATTCAAGCACACAATAA
ATCAGCTCACTTCCAAAGTTTTGTAGCATTTGCTCAAACTGGCGTACTTGTAGCACCTTTAGATGTAAAAACATTACAAGGTTAATCATAACTTCTTTGAAAG
GATGACGCATCATGAATGAAGCAATTCGTACAATTCAAGATCATCGTTCTATCCGTCAGTATACAGACGAAGCAGTAAGTGACGAACATTTAGATACAATT
ATTCAATCTGCTCAGTCAGCCGCTTCTTCTATTAATGGACAGCAAGTAACTATTATTAGCGTCCAAGATAAAGAAAAGAAGAAAAAACTTTCAGAGCTTGCC
GGCAACCAAGCGTGGATTGACCAAGCTCCTCTATTTTTAATCTTTTGTGCAGATTTTAATCGTGCTAAGATTGCAGCTGAACTTAACGATGCCCCTCTTGGCG
TAACCGATGGACTTGAATCAATTTTAGTCGGGGCAACTGATGCTGGAATTTCTTTAGAAGCCGCAACAGTTGCAGCAGAATCACTCGGTTTAGGTACAGTC
CCAATCGGAGGAATTCGCAGAAAGCCGCTAGAAGTCATTGAACTGCTCGATTTACCGGAATACGTATTCCCAGTAAGCGGTCTTGTGGTTGGACATCCGTC
AGATCATTCTGCGAAAAAGCCTCGATTGCCACAAGCTGCTGTGCACCATCGTGAAAGCTATAATCACGACTTGAAGTCATTGATTCAAGACTATGATGCTGA
AATGGCAGAGTATATGAAAAAGAGAACAAACGGTGCTGATGACCGCAACTGGTCACAAACTGTATCAGCTATTTACAAAACCATTTATTATCCAGAAGTTC
GAGCGATGCTTGAAAAACAAGGATTTAAATTCGAATAACCAAAAGCTGCGATGAACATCGCAGCTTTTTTATCTACTTTAACAGCGTGCTGAATAGACTAA
AAGTAATACTCAATAAATGAGGTGTTGATATGGAGTTTATTTTAATTTGTAATTTTCTTTTAAGTGCAACCGCAGCGTTTTTATTGGTGCCGGCTTTTACAAA
AAAGCACACACTTCACGTAACTTTACTTTCTCTCCTTTTCCTCCTTTCGATTGTCCCGACTTTTCTTCTCGCTAATCCACATATCGGCGTTTTGCCTTGGGCTATT
CGAGACGGTCTTCTATTGTTCGCCGCTTTCCTTTTCTTTTATTTAACCTTCACTTTAAACAGATGGAAAAAGCTTTTGTTTTTTCTTCTATTTTTACTGTCGATTG
CTCCTCTTTCCTACGTCGTATATGACGACTTCACGCATACGTATATAGATGCCAATATTGGGCTTGGCCTAGGCTTTTTATTGACATGGCTCTTAACAGCTCT
GTACAATATTTTGGCTATTCTTCGATTTTTTCGTCAGAAATCTCAGTAAACATATCAAAAGTTAACAAACAAACGAATAGTTGATTTTATTTAGTGTACGCTA
ACAACTGCACAGTCCTTCTCTTTACATAAAGCTGTGTATACTAAAAAAGGAGTGATACTTGATGAAACGACTATTCGTAATGGCAGCAGTTATTGCCTTCTT
GGCACTTAGCCGCATGTTAGTATTTGCCGCTGAAACACCTCCTGCTGCAGATGAGCGAACAGCTCAAAATGCAGAAGTTCGCATCGTTCATGCTTCACCGG
ATGCTCCGGCTGTAGACATTTTTGTGGATGACAAAGCTTTAGTAGAAGGAGCAAAGTTTAAAGATGTCTCCAACTCCTTGCCTTTGTCAGCCGGTTCTCATA
AAGTAGAAGTATACGAAGCGAAGACAAAAGGAACAAAAGATTCCATCATCGAAGCTACCCTTGTTGTAGACGGTGGAAAATCGTATACAGTGGCCGCTGT
AAATAAAGCGGAAAATCTTGAACTACAGGCTTTCACGAATAATAAGCAAGGAATGGATGGAAAAGCAAGCTTACGTGTTGCTCATTTATCTCCTGATGCTC
CTGCGGTAAACGTAGGTCCTAAAGGTGCTGCTCCTTTATTTAAAGAGCTGTCTTTTAAAAGCATTTCAGGTTATCAAGTAGTAAATCCGGGTTCGTATGATC
TATCTGTTTCCACAGCAGATGGAAAAGAAATACTAAGTCTTCCTGGAACAAATCTTCAAAGCGGTAAAAACTATACGGTTCTCGCAGTAAATACTGCTGACA
AGCTTGAGACCATTGTTTTACAAGATAATTAATTAAAAAACGTCATTTCAATTGAAATGACGTTTTTTATAGACTGCGCTGAATGAATTCTTCTTTTTTGTACT
CATAGGTAGAAATATCGTTCCACAATCGTTTTGAAAGCTCGTATTTCATACCTTCATATTTTTTTCTAGCACGTTCGCTCGAGCGTAACTGCTCCCTGAAATTA
ATTCGTTCGTTCCAGTAAGGAGATTCACTGACCGTTACATACACATGACTTCTTCTAGCATCATGTGGAATGAGCCTACGGGCATTTTTAGCTGTCATAATTG
GAGGATAGTGTGCAGCTGGAACGCCTTCTTTTAATAAGACAAACATCTCTTTTTCCGCTACATATACGTGAGATTTATTTTTTAATATAGCCCGCACTTCATTT
ATGTCTTCTTCGCGTTTGACTCCGATTAAAATATCAACGATTGGTGAACCTGCCAATCCTGGAACGGACGTGCTTCCAATATGTTCGATGTCTACTGAAAAG
TCTTTTAGTGTATTAAGCAACGCTTCTTTTTCCATTTGATACAAAAGCGACCACTTTGTTTCATACTGAACAATCTTTCGCATATCAGTTTCCACCCTTTCACTT
TTTTATACTGTATCATGTTTCAAAATAAAAAGAAAGAATTTTCTAACTATTTAATTTTAGTTTATGCTACGAACTTTACAGCAATATAGACAGGCTTTTTATTA
TTTTATATATTTTTGTTATTTCTATTCTAAATGTTTCATAATTGTGGATGTTTCCTCCTCCAAAAGATAGATACGATTTTATCTGCTATCTCTCCTCTATCCTATT
CACATCCGGCTGCTTTATACTATTTTACATTTTTTCTAGTATAAAAAACGTCCTCAAAATGTCGATAAACAAAGGACTAAATGCTTTTAATATCTCTATATTCC
ACCTTTAACATAATTATATTTTACATTAATGTAAAATTTTATCGGTAAGGATGGCAGATAGTTCAAATTCATTATACAATTGAATTTCGACATATAGTATGAT
GGATAATATCGGTATTATGAGATAATAGTAGATTTCACTCAAAATTTTGGACTATTTGCCGATATATGCTTTGTATACCAATAACCTTATGTTGAAATTTGTA
ATTTGCAAGGAGGAAAAAATGAGTAAAAAACATGTCATGTACACAGCCGTTTCTTCTGTTCTTTTAAGCTCTTCATTGCTTTTGCACTCAATTGATGCAGATG
CAGCTGAATCGGCCAAAGTAACTGCTACATCGTTAAATGTACGTGCAACGCCTAGTACAAGCGGTGCTATCGTTGGAAAAATAACGAAGGGAAACACCGT
TGATATAGTAGATGAAAGCAAAGGATGGGCTAAAATTACCTATAGCGGTAAAGAAGCTTGGATTTCATCTCAATACATAAATAAAACGCAAACAAACAGTA
CGTCTACCGCAAATTCGACATCGAAGTCAGCGGTCGTTAATGCGAGCTCATTAAACGTTCGTTCTTCTGCAAGCACAAGCGCATCCATCGTGACAAACTTAC
CGCGCAATTCAAAAGTAACGGTGGTAAAAGTAAGCGGATCTTGGTCGCAGGTTAAAACGGCATCGGGTCAAACCGGCTGGGTGGCGAGTCAGTACTTAC
AAGCAGGCTCTGCTTCTTCTGCGCCAGCTAAAGATTCTGGGTCCACATCATCACAGTCAGCGGTCGTTAATGCCAGTTCATTGAACGTGCGCTCTTCTGCAA
GCACAAGCGCGTCCATCGTAACGAACTTACCGCGTAATTCAAAAGTAACGGTGGTAAAAGTAAGCGGATCTTGGTCGCAGGTTAAAACGGCATCGGGTCA
AACCGGCTGGGTGGCGAGTCAGTATTTACAAGCAGGCTCTGCTTCTTCTGCGCCAGCTAAAGATTCTGGGTCTACGTCATCACAGTCAGCGGTCGTTAACG
CGAGCTCATTAAACGTTCGTTCTTCTGCAAGCACAGGCGCGTCCATCGTGACGAGCTTATCGCGCAATTCAAAAGTAACGGTGGTAAAAGTAAGCGGATCT
TGGTCGCAGATTAAAACGGCATCGGGTCAAACCGGCTGGGTGGCGAGTCAGTATTTAAAAGCTGATTCTGGACAGTCTTCACAACCAGCCCAGTCGATTCA
AATTACAAAAGCTTCAAATTTGCGCAGTCAGCCGAGCTTAAGTGCGGGAATCATTCGTGTAGCAAAAGCAGGTGAACGATTCAAAAAAGTAGGCGAAACG

AATGACTGGGTGCAAATTCAGTACTCTGCAAGTCAAACAGCATGGGTGTCAAAAGGGTTAACAGCTGCGGCTGGTTCTACAACTTCAGCTAGTTTGCCTTC
CTCATCTGCTGCTTTAAAAGATAAAATTATTGTAGTAGACGCTGGGCACGGCGGATATGATCCTGGCACTTCAGGCAAGTCATCGATTGAGAAAAATTTAG
CGTTAACAACAGCAAAACTTGTGGCAAGCCGCCTAGCTAATTCTGGAGCAAATGTATTTATGACGCGTTCAAACGATACTTTTATCTCACTAAGCGGACGAG
TGAACGTTAGTGAAGCAAAGCATGCAGATGCATTTATCAGCATTCACTATAACGCTTCTACTTCATCTTCTGCAAACGGCATCGCATCATTTTATTACAGTGA
AGCCAAAGATAAAGAATTAGCAAAATACATTCAAGAAAGCATGGCTGAAAACGCGCCTTCTATGAAGGATATGGGGGTTCGTTTTGGAGATTATTATGTTA
CTCGAGAAAACAACCAGCGTGCTGTACTTCTTGAACTAGGCTTTTTATCAAATGCTCACGATGAGCAAATTGTTTCTTCTAACGCGTATCAGCAGCAAATAT
CTACAGGTATTTACCAAGGAATTGCTCGATATTTTGCAGCTAATAAATAATAAAAGCCCTCATAATGAGGGCTTTTATACTACCAGCTTGCTTTTTTAACACC
TGGAATTTGTCCTTTGTAGGCCAATTCCCTAAAAGCGATGCGTGACATTTCAAATTTTCTAAGATAGCCTCGCGGACGACCGGTAACACGGCAGCGATTATT
TAAGCGGGTTGGTGAAGAATCTCTAGGCAATTTTTTTAACGCTTCGTAATCTCCTTTTTCCTTCAGCTCTTGGCGTAATTGCGCATATCTCGCTACCATCTCTT
GACGCTTTTGTTCTTTCACAACTTTTGATTTTTTAGCCAAACTCTTTGTCATCCTTTCGTTTTATCAATTTTTTATTTGAAATACGTAATCATTACGATTTAATTA
TTTTGAGCAAGTTACTTTCTAAATTTAATCATATTTTCTTCATTTTTTCAACTTGTTTGCTTCCATGCTTACATGTAGCCTACCCCCAGGCTGCTGCATAAACAT
AAAAAAAATTGTATGTCAAATGTAATTAATTCCTGCATCTCCTATATCATACAAAGAGCCCTACATATTCCATATTGTAGGAGGATTTGTATTTACAGATAAT
ATTTCAATAATATTACAGTTCATTTACATATTAATAACTTCGACAAATTTAGCGGTTATCTAGTATATAATCATCTTAGAAATTCCAAAAATAATATTTTGCGT
ACTCAATAAAATATAAAAGTAAAGGTGATTAAAAAATGAAATCGACTGGTATTGTAAGAAAAGTAGATGATTTAGGTCGTATCGTAATTCCTAAAGAGTTA
AGACGTGTTCTTGAAATAGATGTAAAAGATCCTGTTGAAATTTTTGTCGAAACAGATCGAATTATTTTAAAGAAATATGAGCCGAACGGTGCTTGTGCGATT
ACTGGCGAAGTATCTTCTGACAACGTAACAATCGCAGATGGCAAATTAACTTTAAGCCGCGAAGGTATGGAGTTATTACTTGAAGAGTTAAAACAACGTAA
TTTCTAATAAAAAGAAAAGCAGCTGCTCATATGCAGCTGTTTTTCTTTTTGTTAAAGTTTTTTCTTCGCTTTCTGAGTTTCAGATGCTATACTCAGAAGCGAAA
TACATAATAAAGGAGCCTTTCATCATGACAGTTGAACAAATTACTCATCACTTTTTAGCACATCGAAACGTAACTAATGAATTAATCAACAAAATTGAAGCA
GGTCACTACGAATACAAGCCTGCCCCGACATCTATGTCCGCTTTTGACTTATCTACGCATATTTTAACATCTTTTTATGCATTTGCATCAGTGGTAAAATCCGG
TGATTTATCTCCACTTGGTCAAAAAGCTGATTTTAGCGGTAAATCATTATCGGACCTTGCAGCTGAATATACGCAAAAAACAGTAGACGTATTACGTTCACT
TAGAGAAGATGATTTAAACAAAACAATTGACACCACTGCAGCGTTTGGAAGAAAGCTTCCCGGAAGTCAACTCTTACAAATGGCCATTGACCATGAAATTC
ATCATAAAGGAAATTTATACGTCTACGTACGTGAGATGGGTCATACAGATCTTCCTCTATTTGTTCAGTTCGGTTAATAAAATAGTTCGTGCTTGTAAGCGCC
CAAGCTAGAAGTTTGGTGCGCTTTTTCTTTGCAGTTTTCGGCATAATAAGTGAGATATATGGTATAATTCTATCAATAACTTCATATCTACTATTCAAAGCTT
GTTTCTTCTTATAAAACGATAGTCATCATCTTTTACAAAGGAGGAATCACTTCATGAGTCAAACTGTTAATCAACAAAAAATTGCTCAAGTGTATCATCTTCC
GTTTGTTTCTTCATTTGGAGGAAGTGCTATGAACCTTCTTTCTCCTCAAGCTGGCGAACATATTTTAGATATTGGCTGCGGCACTGGCGAATTTATTTCGCAG
CTCAATAAGCTTCATGTCCGTGCAACGGGCATCGATTGCTCTGAAAATATGATTGAACAGGCTCGTCATCAGTACCCAACTTTATCCTTTGAACAGTTAAAT
GCCCTTAACCTTCCTTATTACGAACAATTTGACGCAGTTTTTTCAAATGCAACTTTACACGACATCCGTCCCCCTCACCTTGTCATTAAAAACATTTACAATGC
TTTAAAACCAAATGGCCGATTTGTAGCAGAATTTTGGGGAAAAGGAAACATGGACGAAATTTTCACAGAAATCATTCGTCAATTTAAAACGTTTGATATCCC
TTATAACGATGTAGATTTCCCATGGTACTTTCCTTCTGTAGCTGAATATTCTTCTCTTTTGGAATCACAGAATTTTCAAGTCACCACGGCTATTCACTACAGCC
GTCCCTTCTTGTTAAAAGGGGAAAACGGCTTGCGTAATTGGCTGGAAACAAATGCAGCCCCTCTTTTTGCTCACGTGACGACAGAAACTAAAAATGTCCTC
ATTACGCGCTTAGAAAATCGAATGCAAGATAAACTATTTCAAAATGGATCTTGGCTAGCAGAAGCTAAACATATACGTGTACATGCTGTAAAACGAGATTG
AAAAATGGACGTGATTCTATTCACGTCCATTTTCGTTAAAGTGCAGCCAATATCCTTTTCCTCGAATCACTTCGATTTCTAAATATGTTCCTATTTTTTTGCGA
ACCCGGTAAATCAGCGCATTCAGCTCTTCCGCTGCTACTAGAATTTCTCCATTTGACTCTACTCGTTCAGGCCAAACGCTTTTTTTTAATGCTTCTTTTGAAAC
AAATTGATTGTTGTATTCTAGAAGATAAGCTAAGCACGCGTATTCTTTATCTGACAGAATATACGTTTCCTCTTTTATTTTTACTTTCTGCAAAATTGGATCTA
ATGTGTAAAATGGCTTATTTATTTGTTTTAGCAAAGGTCCTAAATCGAGGGTTGCATCCATATCATCATTTCTAAATACTAAAGCTGCCTCTTCATTTGCTAAG
GTAATGACATCCTGCTCTTTCAATAAACGAGGGGTTCCAGGCTGTAACTCTTCTCCATTTACTTTTGTCCCGTGTTTACTAAACAAGTCTGTAATGAAAAAGG
CATTGCTTTCGTACGTTACTTCAAAATGCTTGCGCGAAATGTAAGCACTTTGAAAGGCCAGATAAGGCTCCCATTCTTCACTGCTTCGTCCGATAATACAGG
CTTTATCTTTTGGAATAGGCACGGAAGCTCCTTCATAAAAAGGAGCTCCGTTCCGAATAATCAACATACCGTTCATTGTTTGCTCACACTCTTTTTTAAAATTT
TCTGACTCTTATCATGTAATTATAACACTCACATTTATTGAGATTTCTTCCAACTTTGGATAACATCATAGTATAGTCAGTTATATGATAAAGGTTGTTGCCAT
GTAAGTAAACCTTTAGAGCAGTCCTTTTCTAACTGAAAAAATACGTATTCTTTGCCCTGCTGTAATGGGGATACAATTTTTTTGTAAAATAATTTAGTTTTTAG
TAACTTTGACATATGTTCTCTCTTTTTAAAGCGGAGTAAAATAGGAAAATACGTGAAATATTTACATAATGGAGGAAGACCAATGGGACGTTTGCCAAAAG
ATAGAGAAATTCAAGCGATTAAACGACGCGTTGCACTGGTATTGTTAATTATTGGATCATTTATTTTAACAGGTGCTAGCTTTAGCATTTATTGGTTTATGAT
GTTAAAAATATAAGATTTTTTTATAAAATAAAGAAAAAAGACATGGTTTTGTTTCCATGTCTTTTTTAGGTTTATTCTTTCTCAGACACTCCAGCAGATTGAAA
CGTAGCCATATTGGCAATCATCAGTGAAGCCGAGTGCAGCAGCGGAAATGTAACAGCAGCTCCACTTCCCTCACCTAAACGAAGATTGAGGTTGAGGAGC
GGTTTCTTGCCAAGAAGCTTCATTGCACACGCATGGCCTTGTTCAACAGATTCGTGACTAGCAAACATATAGTCTCTTGCTTGTGTATTGATTTCTGTTGCTA
GAAGGGCTGCAACTGTACAAATAAAACCATCTACTAAAATAGGAATACGGTGAGCAGCTGCCGCCAGCATAGCACCTGTCATCCCGGCAATTTCAAGACCC
CCTATTTTACTAATAATATCAATCGGATCCTGAGGGTTAATGTGCCTGTTTTGCAGCGCTTCTTCAATCACGTTCACTTTGTGGCGCAGTTGTTCATCAGAAA
TCCCTGTGCCCATTCCCGCAAGCTTAGAGACTTTTTCTCCTGTCAATACAGCTAAAATAGCCGTGCTTGACGTAGTATTTCCGATGCCCATTTCTCCTACAATC
AAACATTTAATTCCTTGGGCAATAAGCTCTTCAGCTTCTTCATATCCGACTTGAACAGCCAGTTCTGCCTCATTGCGCGTCATCGCGTCTTCTTTATAAAAACT
TGCTGTCCCCAGCTTCACTTTGCGTTGTACAAGGTCCTTATGGGAAAGGTCAGAAGCAATGGGCC
