# Supplementary material for: Counterintuitive DNA Sequence Dependence in Supercoiling-Induced DNA Melting
Source: PLoS One. 2015 Oct 29;10(10):e0141576. doi: 10.1371/journal.pone.0141576 (PMC4625975; doi:10.1371/journal.pone.0141576)
Supplement: S1 Table — Sequence of the primers used to PCR the 10kb DNA fragments which were used to clone into pCR-XL-Topo vector. All PCR’s were performed using KOD Xtreme polymerase (MerckMillipore). To be able to make the 77% GC construct we first developed two PCR’s ~5kb, which were cloned into pSuperCos1 (stratagene), using restriction enzymes specified (partially sequence verified). From this plasmid the 10kb PCR was developed and cloned into pCR-XL-Topo vector. (PDF) [file pone.0141576.s007.pdf]

Construct	Fw primer	Rev primer	Restriction enzymes	Template DNA
**1) 77% GC content**	**TTTTGCGGCCGCCCAGGCCCACCCCCTCGTGGA**	**TTTTCTCGAGGGGACCACAAAGGCCAGAATTCACGGCTG**	**NotI + XhoI**	pSuperCos1-Streptomyces Clavuligerus DNA
pSuperCos1-Streptomyces Clavuligerus DNA	**CACACAGCGGCCGCCCAGGCCCACCCCCTCGTGGACGAG**	**CACACAGGATCCGGCTTCCGCCCGGTGCGGCTCAGTCAC**	**NotI + BamHI**	Streptomyces Clavuligerus DNA
pSuperCos1-Streptomyces Clavuligerus DNA	**CACACAGGATCCCCGGGTGTCCTCGGCTTCCTG**	**CACACAGAATTCACGGCTGAACTGGGTTCTCTCCCG**	**BamHI + EcoRI**	Streptomyces Clavuligerus DNA
**2) 58% GC content**	**TTTTGCGGCCGCACAGTAATTACGGTGCTGCGCTGGAGAAACAGGG**	**TTTTCTCGAGCGCCGTTAACCCGGACGTGCTGA**	**NotI + XhoI**	Unmethylated *c*I857 *Sam7* Lambda DNA
**3) 42% asymmetric spread of GC content**	**TTTTGCGGCCGCTCCGTGGTGGCACAGAGTACGGCAGA**	**TTTTCTCGAGAGAGTTTCTGCGGCAGTTAATCGAACAAGACCCGT**	**NotI + XhoI**	Unmethylated *c*I857 *Sam7* Lambda DNA
**4) 42% GC content**	**TTTTGCGGCCGCCGAAATTTTCTTCATAATAAAAGTTAAAAGCCGCATCTCTCGCTACAGC**	**TTTTCTCGAGAAGTGACGGAACTGGATTTGGATGAAACGGTTCATACAACGC**	**NotI + XhoI**	Bacillus Megaterium QM B1551 DNA
**5) 39% GC content**	**TTTTGCGGCCGCGGAAATCAAGATGAACTAATTTATACGTACAAAGTAAAAATTGGGAACA**	**TTTTCTCGAGTTTGCATAGTGCACAATTAAATCGCGATTGTCCGCGG**	**NotI + XhoI**	Bacillus Megaterium QM B1551 DNA
**6) 38% GC content**	**TTTTGCGGCCGCCGCTAAACACTGTACAATTCGCAGCCGGTTAGGATCA**	**TTTTGGGCCCATTGCTTCTGACCTTTCCCATAAGGACCTTGTACAACGCA**	**NotI + ApaI**	Bacillus Megaterium QM B1551 DNA
